# A Smartphone Intervention to Promote a Sustainable Healthy Diet: Protocol for a Pilot Study

**DOI:** 10.2196/41443

**Published:** 2023-03-02

**Authors:** Ujué Fresán, Paquito Bernard, Sergi Fabregues, Anna Boronat, Vera Araújo-Soares, Laura M König, Guillaume Chevance

**Affiliations:** 1 ISGlobal Barcelona Spain; 2 Department of Physical Activity Sciences Université du Québec à Montréal Montréal, QC Canada; 3 Research Center University Institute of Mental Health at Montreal Montréal, QC Canada; 4 Department of Psychology and Education Open University of Catalonia Barcelona Spain; 5 Integrative Pharmacology and Systems Neurosciences Research Group Neurosciences Research Program Hospital del Mar Medical Research Institute (IMIM) Barcelona Spain; 6 Center for Preventive Medicine and Digital Health (CPD) Medical Faculty Mannheim Heidelberg University Heidelberg Netherlands; 7 Faculty of Life Sciences: Food, Nutrition and Health University of Bayreuth Bayreuth Germany

**Keywords:** eating behavior change protocol, sustainable diet, dietary sustainability, eating behavior change, n-of-1, nutritional education, eHealth, mobile health, mHealth, mobile phone

## Abstract

**Background:**

Changing current dietary patterns into sustainable healthy diets (ie, healthy diets with low environmental impact and socioeconomic fairness) is urgent. So far, few eating behavior change interventions have addressed all the dimensions of sustainable healthy diets at once and used cutting-edge methods from the field of digital health behavior change.

**Objective:**

The primary objectives of this pilot study were to assess the feasibility and effectiveness of an individual behavior change intervention toward the adoption of a more environmentally sustainable healthy diet as a whole and changes in specific relevant food groups, food waste, and obtaining food from fair sources. The secondary objectives included the identification of mechanisms of action that potentially mediate the effect of the intervention on behaviors, identification of potential spillover effects and covariations among different food outcomes, and identification of the role of socioeconomic status in behavior changes.

**Methods:**

We will run a series of ABA n-of-1 trials over a year, with the first A phase corresponding to a 2-week baseline evaluation, the B phase to a 22-week intervention, and the second A phase to a 24-week postintervention follow-up. We plan to enroll 21 participants from low, middle, and high socioeconomic statuses, with 7 from each socioeconomic group. The intervention will involve sending text messages and providing brief individualized web-based feedback sessions based on regular app-based assessments of eating behavior. The text messages will contain brief educational messages on human health and the environmental and socioeconomic effects of dietary choices; motivational messages to encourage the adoption of sustainable healthy diets by participants, providing tips to achieve their own behavioral goals; or links to recipes. Both quantitative and qualitative data will be collected. Quantitative data (eg, on eating behaviors and motivation) will be collected through self-reported questionnaires on several weekly bursts spread through the study. Qualitative data will be collected through 3 individual semistructured interviews before the intervention period, at the end of the intervention period, and at the end of the study. Analyses will be performed at both the individual and group levels depending on the outcome and objective.

**Results:**

The first participants were recruited in October 2022. The final results are expected by October 2023.

**Conclusions:**

The results of this pilot study will be useful for designing future larger interventions on individual behavior change for sustainable healthy diets.

**International Registered Report Identifier (IRRID):**

PRR1-10.2196/41443

## Introduction

### Background

Sustainable healthy diets have been defined as healthy dietary patterns with low environmental impact and being economically fair and affordable and culturally acceptable, which contribute to nutrition security and a healthy life for the present and future generations [1]. Such diets are characterized by being nutritionally balanced patterns, mainly (if not totally) based on unprocessed or minimally processed plant-based foods obtained from fair sources and with minimal food waste [2]. Over the past decades, the way food has been produced and consumed is far from being healthy or sustainable: unhealthy diets are the leading cause of morbidity and mortality worldwide [3]; healthy diets are not affordable and accessible to a large proportion of the world’s population [4]; the current food system is among the main drivers of environmental pollution and natural resource use [5]; and from the socioeconomic and ethical perspectives, there are countless cases of forced labor conditions and unfair salaries across the food system [6]. Altogether, changing the current food system toward a sustainable and healthy one is timely. This mission requires large transformative changes in each food system phase, from production to consumption, and all stakeholders, from producers to consumers, should be involved in such a transformation [7].

To encourage the adoption of sustainable healthy diets, structural changes (ie, political actions or nudges) have to be combined with strategies explicitly tailored to individual behavior change [8,9]. To date, most individual eating behavior change interventions implemented have mainly focused on improving health and, to a lesser extent, on the dimensions of sustainable eating behaviors (ie, their environmental impacts and, with even less consideration in the literature, their social consequences [10]). Evaluating all these dimensions at once within interventions is crucial because they are not necessarily aligned. For instance, fish is a healthy food but could be obtained from overexploited fish stocks, exacerbating environmental damage [11]; even if captured from sustainable sources and using environmentally responsible techniques, labor conditions for fishery workers may not be fair, compromising their well-being [6].

### Prior Work

Recently, some interventions have been designed with the aim of promoting individual eating behavior change considering different dietary sustainability dimensions. For instance, O’Sullivan et al [12] have designed an intervention to encourage the adherence to a healthy diet with low environmental impact, but they have left aside other relevant behaviors to achieve a sustainable diet, such as food waste, and the socioeconomic consequences of dietary behaviors. An intervention implemented by Monroe et al [13] among US students has targeted food waste reduction and considered the social dimension of individual eating behaviors, such as promotion of the acquisition of local foods from farmers’ market. However, this intervention has not tackled the overall dietary pattern of the participants, focusing only on red meat consumption as the main outcome. Besides considering red meat reduction is key to transitioning to sustainable healthy diets, this approach lacks an assessment of the substitution effect and possibility of other changes in untargeted food groups (ie, spillovers), which could offset the overall effect of the intervention [14]. For example, if pork meat is replaced by hard cheese (one of the food products with the highest environmental impact), the dietary environmental impact would increase [15]. Therefore, eating behavior change interventions aimed at promoting sustainable healthy diets as a whole, that is, as a complex interplay of behaviors, are needed [16].

Beyond interventional studies, reviews have documented potential mechanisms of action that motivate or impede behavior change for a healthy and sustainable diet. A diversity of personal and interpersonal factors have been highlighted, such as sociodemographic determinants (ie, gender, age, socioeconomic level, and political orientation), food-related factors (ie, healthiness, taste, and affordability), generic motivational factors (ie, self-efficacy and habit), and specific motives for sustainability (ie, environmental impact, ecoanxiety, and social justice) [17-21]. By contrast, specific barriers have been identified, such as perceiving sustainable healthy food as less tasty [19]. These previous findings are useful for designing interventions (as levers to manipulate and be considered during a study) and for understanding the mechanisms of action that potentially mediate the effect of an intervention on behavior change.

At the methodological level, specific limitations of previous eating and proenvironmental behavior change interventions have also been repeatedly mentioned in the literature. They include the use of long, paper-based food frequency questionnaires that do not allow for frequent and dynamic assessment of the behavior change process [22], short or nonexisting follow-up periods that preclude the appreciation of long-term changes and potential forms of behavioral maintenance over time, and study designs inappropriate for testing causal relationships [23]. To build upon previous research, this pilot trial has been designed to promote individual behavior change for a sustainable healthy diet over a year with measurement bursts of daily eating behaviors digitally implemented through smartphones to better understand and monitor the behavior change process through the study [24].

The primary objectives of this study are as follows:

1.1 To evaluate the effectiveness of a pilot digital behavior change intervention promoting a more environmentally sustainable healthy diet (1.1.a) and relevant specific food outcomes related to dietary sustainability (1.1.b)

By sustainable healthy diet, we refer to a healthy dietary pattern with low environmental impact (objective 1.1.a), that is, incentivizing the consumption of fruits; vegetables; legumes; nuts; whole grains instead of their refined versions; unsaturated and nonrefined vegetable oils as main dietary fats; and water as the main beverage instead of others, such as sodas or alcoholic drinks, while promoting a reduction in the consumption of meat (especially red and processed meats), dairy products (especially cheese), sugar-added products, products high in salt, and ultraprocessed foods [25], with minimal food waste and the purchase of food from fair and ethical sources.

Specific food groups or relevant food-related outcomes that will be investigated closely (objective 1.1.b) include: the consumption of red and processed meat, fruits and vegetables, ultraprocessed foods, and dairy products, and food waste.

1.2 To examine the feasibility and acceptability of the intervention and collect relevant information for iteratively improving future interventions

The secondary objectives are as follows:

2.1. The identification of mechanisms of action, notably motivational ones, potentially mediating the effect of the intervention on behaviors

2.2. The identification of potential spillover effects and covariations among different food groups (eg, meat, fruits and vegetables, and ultraprocessed food) and dietary outcomes (ie, diet composition and food waste) occurring over the study

2.3. The identification of potential interaction effects between socioeconomic status and intervention effects on behaviors

## Methods

### Participants

Potential participants will be approached on the web via email and social media with the support of our institutional communication and outreach activities team (ie, Barcelona Institute for Global Health, ISGlobal). They will be invited to visit a website with a brief description of the study, which will contain a link to a Google form (Google LLC) with questions related to our inclusion and exclusion criteria (provided subsequently) that is to be completed by those interested in participating. Those meeting the inclusion criteria will be classified in 3 groups according to their subjective socioeconomic level. The reason behind this stratification is that socioeconomic level is a major determinant of food choices and individual dietary carbon footprints [26,27]. In addition, deprived populations might benefit less from interventions delivered via digital or mobile means; therefore, it is necessary to include social inequality indicators such as socioeconomic status in the evaluation of interventions to assess their potential differential impact [28,29]. We chose a subjective measure of socioeconomic level and status because it has been shown that socioeconomic status has an impact on how individuals perceive resources beyond their factual resources [30]. This indicator is also strongly associated with physical functioning and health outcomes, beyond objective measurements of socioeconomical level [31]. The MacArthur Scale of Subjective Social Status will be used [32]. This scale ranges from 1 (“perception of low social status”) to 10 (“perception of high social status”). In our study, participants scoring themselves from 1 to 3, 4 to 7, and 8 to 10 will form groups of low, moderate, and high socioeconomic statuses, respectively. Within each group, the first 7 participants meeting the inclusion criteria will be invited to participate in the study after stratification by gender, with the aim of having a balanced number of individuals identifying themselves as women and men in each group. In total, we aim to include 21 participants. The questionnaire used for checking the inclusion and exclusion criteria is provided in Multimedia Appendix 1.

Individuals will be enrolled in the study if they meet the following characteristics: adults aged from 18 to 65 years; residing in Barcelona; speaking and reading Spanish fluently; having a mobile phone supporting the installation of a smartphone app; reporting no history of eating disorders (eg, anorexia nervosa, bulimia nervosa, and binge eating) or any food intolerance or allergy (eg, celiac disease, and nut allergy) or other chronic illnesses that might directly impact eating behaviors (eg, diabetes, cardiovascular disease, cancer, and obesity); following an omnivorous diet; and scoring ≥5 out of 10 points on the dietary questionnaire included in the Google form (refer to Multimedia Appendix 2 for the scoring criteria). The exclusion criteria are being pregnant or planning to be pregnant in the next year, being in a postnatal situation for <3 months, breastfeeding, being professional athletes, following specific diets (eg, vegan, vegetarian, high protein, slimming, gluten free, and low in sugars), reporting any illnesses that might directly impact eating behaviors, and not taking their own decisions about food choices (eg, someone else, such as husband or wife or partner or mother or father, selects what they eat for most of their meals).

Participants will receive financial incentives for their participation. Over the course of the study, there will be 15 evaluation weeks, during which some questions should be answered daily and others once a week (refer to *Measurement* section). For each evaluation week, they will receive €10 (US $10.7) if they complete at least 6 out of the 7 daily questionnaires plus the weekly assessment. Thus, in total, each participant can receive €150 (US $160.4) for full participation in the study. At the end of the study, they will receive their money through bank transfer.

### Study Design

**Figure 1:**
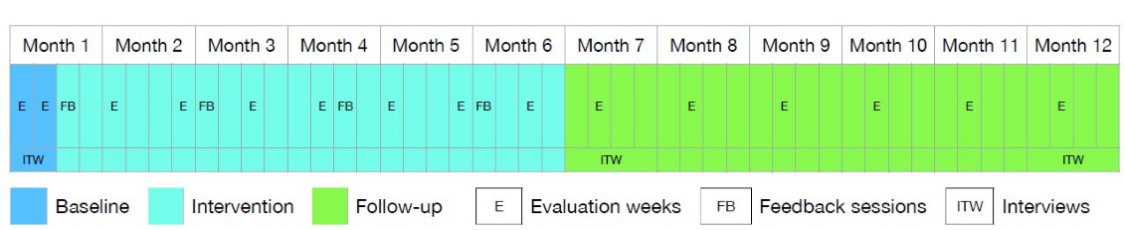
Study timeline.

We will run a series of 1-year long ABA hybrid n-of-1 trials, with the first A phase corresponding to a 2-week baseline evaluation, the B phase to a 22-week intervention, and the second A phase to a 6-month postintervention follow-up (refer to Figure 1 for an illustration). We labeled the trials as “hybrid” because, in contrast with pure n-of-trials, we will conduct both individual and group-level analyses depending on the hypothesis tested and for maximizing statistical power, as has been done in similar pilot studies previously, for example, the study by Korinek et al [33]. Therefore, we did not rely on the available checklists for n-of-1 trials, such as the SCRIBE (Single-Case Reporting Guideline In Behavioral Interventions) guideline [34]. In addition to the n-of-1 trials, semistructured interviews will be conducted with the participants to collect contextual information that may aid in understanding the mechanisms by which any individual changes in the participants’ outcomes may have occurred, complement the quantitative findings on the intervention’s feasibility, and assess the intervention’s delivery and acceptability.

We labeled this study as a pilot study because our intention is to generate pilot data to plan further iterations of this intervention in the future and build upon this previous experience. Hence, this study aims to produce generalizable knowledge and cannot be labeled as a full trial; however, benefiting from the within-participant measures, testing the effectiveness of the intervention on some specific outcomes and for some specific objectives will be possible.

### Intervention

The intervention will involve sending text messages and providing brief, individualized, web-based feedback sessions based on the eating behavior assessments completed during the study. The text messages will be distributed on 2 nonconsecutive days per week, that is, Tuesdays and Fridays, through the General Data Protection Regulation–compliant app *m-Path* [35] (the same app will be used for implementing the evaluations; refer to the next section, *Measurements*). An in-person meeting with each of the participants will be set on their first day in the study to help them install the app on their mobile phones and provide training if needed. The text messages will contain brief educational messages about human health and the environmental and socioeconomic effects of dietary choices; motivational messages to encourage the adoption of sustainable healthy diets by participants, providing tips to achieve their own objectives; or links to recipes, providing ideas on tasty sustainable healthy meals. These text messages were developed based on a previous attempt to identify relevant behavior change techniques for changing eating behaviors [14] and the compendium of self-enactable techniques to change and self-manage motivation and behavior v1.0 [36].

The understandability and relevance of each message were pretested. Six individuals from our institution matching our inclusion criteria and not implicated in the development of this study were involved in testing and refining the messages. Initially, the messages were tested for comprehension and relevance. Participants rated how understandable each text message was on a scale of 1 to 10. They also had the possibility of commenting on these as well as suggesting improvements for each text message. After this, and based on end users’ suggestions, the text messages were reworded, refined, or even removed. Multimedia Appendix 3 shows the content of the final text messages and how they match specific behavior change techniques.

In addition to the text messages, individualized feedback will be provided via short web-based individual meetings (approximately 15 minutes) at the end of the baseline phase or beginning of the intervention phase and then every 6 weeks during the intervention, after 2 measurement weeks (Figure 1). During the web-based sessions, a member of the research team with extensive knowledge of sustainable healthy diets will review the assessments of the previous weeks with the participants; provide tailored advice to improve their diet with regard to its healthiness and sustainability; and answer any questions, doubts, or additional questions that they may have. No feedback session will be provided during the follow-up period.

### Measurements

Both quantitative and qualitative data will be collected (Table 1).

**Table 1:** Summary table for the study’s objectives and their assessment.

Objective and assessment		Measurement
**1.1.a Changes in the environmental sustainability and healthiness of the diet**		
	Quantitative	Eating behaviors questionnaire
	Qualitative	Baseline interview: Dietary habits Healthy and sustainable diet Postintervention interview: Perceived effectiveness of the intervention
**1.1.b Changes in individual food groups and food waste**		
	Quantitative	Eating behaviors questionnaire
	Qualitative	Baseline interview: Dietary habits Healthy and sustainable diet Postintervention interview: Perceived effectiveness of the intervention
**1.2. Feasibility and implementation of the intervention**		
	Quantitative	Relevance of the interventional text messages
	Qualitative	Interview at baseline: Motivations and expectations from the study Interview after the intervention: Acceptability of the intervention Feasibility of the intervention Elements to improve Interview at the end of the study: Acceptability of the intervention
**2.1. Identifying potential mechanisms of action mediating the effect of the intervention on eating behaviors**		
	Quantitative	Mechanism of action questionnaire
	Qualitative	Interview at baseline: Motivations and expectations from the study Interview after the intervention: Perceived effectiveness of the intervention Feasibility of the intervention Elements to improve
**2.2. Identifying potential covariations and spillover effects among food groups and food composition and waste**		
	Quantitative	Eating behaviors questionnaire
**2.3. Potential interaction of socioeconomic status with the intervention effectiveness**		
	Quantitative	Eating behaviors questionnaire

### Quantitative Measurements

#### Questionnaires

Several self-reported questionnaires will be used to collect quantitative data. With the exception of the questionnaire collecting data about baseline characteristics, the questionnaires will be implemented through the study app (*m-Path*). These questionnaires will pop up as notifications in the app.

##### Baseline Characteristics

A baseline questionnaire will be used to collect descriptive data about the sociodemographic (ie, gender, age, education, household income, political orientation and postal code, number of people they live with, and if they usually cook for others), anthropometric (ie, height and weight), and health-related (ie, self-perceived physical and mental health, supplement intake, and smoking) characteristics of the participants, together with data about their general motivation for protecting the natural environment (Multimedia Appendix 4). It will be completed once at the inclusion through a Google form.

##### Eating Behaviors

As shown in Figure 1, eating behaviors will be assessed daily for 15 weeks through a brief questionnaire: participants will have to report their food intake at the end of each day during the 2 weeks of the baseline period; then, every 3 weeks during the intervention period; and afterward, once per month during the follow-up period, representing a total of 105 potential measurement days per participant spread over a year (15 evaluation weeks × 7 measurement days).

**Figure 2:**
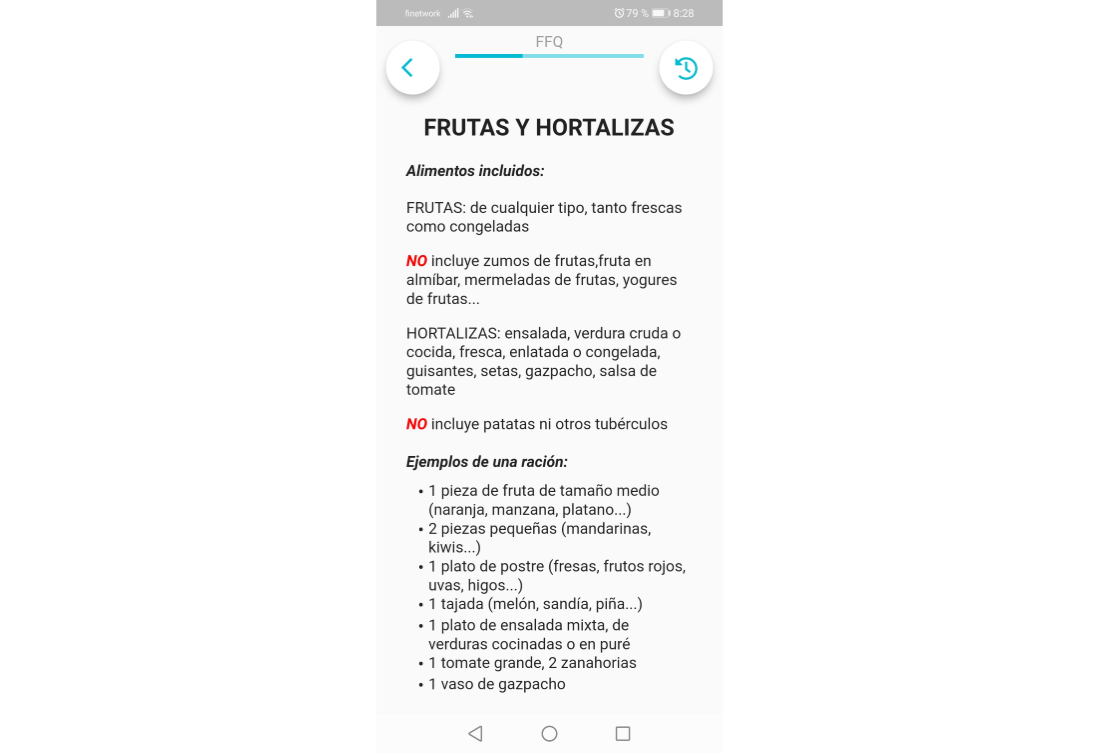
Screenshot of the item aimed at fruit and vegetables consumption.

A 12-item questionnaire will assess the consumption of specific food groups relevant to environmentally sustainable healthy diets as well as food waste (Table 2) [25]. The present food frequency questionnaire was designed considering the need to minimize the total number of items and make it suitable for daily assessment while adding items about relevant food groups to assess food environmental sustainability and healthiness. For each food group, the number of servings consumed during the day will be asked. Specific foods gathered in the assessed food groups will be detailed, and their serving sizes will be provided with examples that the participants can easily interpret. For instance, a serving of vegetable is reported as a medium size dish of mixed salad, cooked vegetables, or vegetable purée—1 big tomato, 2 carrots, and 1 glass of gazpacho (Figure 2). The size of a serving was established based on the Spanish Society of Community Nutrition recommendations and other Spanish documents [37,38]. The exception will be whole grains and virgin olive oil, which will be assessed in terms of their proportion among all the grains and added fats consumed during the day, respectively. The reason for assessing them as a proportion is because of the priority of healthy fat and carbohydrate intake rather than the total amount of fat and carbohydrate consumed, as the recommended amount of total added fats and carbohydrates depends on the specific energy needs of each person.

Beyond food consumption, daily food waste will be measured with a “yes/no” binary question [39]. As, to our knowledge, there are no previous validated questionnaires to objectively assess the social aspect, changes in this specific sustainability dimension will not be quantitatively assessed but will be explored via the qualitative interviews. An extra open-question item will be added at the end of the 12-item questionnaire to give participants the opportunity to provide any comment or clarification if necessary. To ensure that they understand how their food consumption should be reported, participants will be explained at the initial meeting about the quantification of each food group and provided with an explanatory document showing how to respond to the questionnaire (Multimedia Appendix 5). Eventually, this will also be discussed in feedback sessions.

**Table 2:** Items for evaluating food consumption and food waste.

Name and item			Answer options
**How many servings of the following food groups have you consumed today?**			
	Red or processed meat	(1) 0 servings, (2) 0.5 servings, (3) 1 serving, (4) 1.5 servings, (5) 2 servings, (6) 2.5 servings, (7) 3 servings, (8) 3.5 servings, (9) 4 servings, (10) 4.5 servings, (11) 5 servings, and (12) more than 5 servings	
	White meat, fish or eggs	(1) 0 servings, (2) 0.5 servings, (3) 1 serving, (4) 1.5 servings, (5) 2 servings, (6) 2.5 servings, (7) 3 servings, (8) 3.5 servings, (9) 4 servings, (10) 4.5 servings, (11) 5 servings, and (12) more than 5 servings	
	Dairy products	(1) 0 servings, (2) 0.5 servings, (3) 1 serving, (4) 1.5 servings, (5) 2 servings, (6) 2.5 servings, (7) 3 servings, (8) 3.5 servings, (9) 4 servings, (10) 4.5 servings, (11) 5 servings, and (12) more than 5 servings	
	Legumes	(1) 0 servings, (2) 0.5 servings, (3) 1 serving, (4) 1.5 servings, (5) 2 servings, (6) 2.5 servings, (7) 3 servings, (8) 3.5 servings, (9) 4 servings, (10) 4.5 servings, (11) 5 servings, and (12) more than 5 servings	
	Fruits or vegetables	(1) 0 servings, (2) 0.5 servings, (3) 1 serving, (4) 1.5 servings, (5) 2 servings, (6) 2.5 servings, (7) 3 servings, (8) 3.5 servings, (9) 4 servings, (10) 4.5 servings, (11) 5 servings, and (12) more than 5 servings	
	Nuts or seeds	(1) 0 servings, (2) 0.5 servings, (3) 1 serving, (4) 1.5 servings, (5) 2 servings, (6) 2.5 servings, (7) 3 servings, (8) 3.5 servings, (9) 4 servings, (10) 4.5 servings, (11) 5 servings, and (12) more than 5 servings	
	Sugary, salty and ultraprocessed foods	(1) 0 servings, (2) 0.5 servings, (3) 1 serving, (4) 1.5 servings, (5) 2 servings, (6) 2.5 servings, (7) 3 servings, (8) 3.5 servings, (9) 4 servings, (10) 4.5 servings, (11) 5 servings, and (12) more than 5 servings	
	Alcoholic drinks	(1) 0 servings, (2) 0.5 servings, (3) 1 serving, (4) 1.5 servings, (5) 2 servings, (6) 2.5 servings, (7) 3 servings, (8) 3.5 servings, (9) 4 servings, (10) 4.5 servings, (11) 5 servings, and (12) more than 5 servings	
	Sodas, juices, energy drinks, etc	(1) 0 servings, (2) 0.5 servings, (3) 1 serving, (4) 1.5 servings, (5) 2 servings, (6) 2.5 servings, (7) 3 servings, (8) 3.5 servings, (9) 4 servings, (10) 4.5 servings, (11) 5 servings, and (12) more than 5 servings	
**What proportion of the cereals you have eaten today are whole grains?**			
	Whole grains	Scale 0-100: 0=no whole grains at all and 100=all cereals I ate were whole grains	
**What proportion of the oils and fats you have added to your food today is virgin or extra virgin olive oil?**			
	Virgin or extra virgin olive oil?	Scale 0-100: 0=no virgin olive oil at all and 100=all the added fat I consumed were virgin/extra virgin olive oil	
**Have you wasted food today? It includes foods that you discard for different reasons: expired foods, that have gone bad, leftovers both at home and in restaurants...**			
	Food waste	1=yes and 2=no	
**Do you want to comment or clarify any point? If you want to report any food that you have consumed that is not collected in the questionnaire, please, indicate the amount consumed as well.**			
	Comment or clarification	Open question	

##### Eating Behavior Scoring Procedure

In terms of the scoring procedure, we designed an index for assessing dietary healthiness and environmental sustainability for every assessment week based on 10 out of the 12 items provided in Table 1. The item related to white meat, eggs, and fish is not considered in the score to avoid overrating protein groups. However, it will be used in the individual food analysis to assess whether, in the case of a reduction in the consumption of red and processed meat and not a simultaneous increase in legume consumption, red and processed meat is replaced by animal-based proteins (objective 2.2). The second item not included in the present index is the one assessing food waste. This last item will be used as a specific outcome in itself and is not included in our main score.

Therefore, the environmentally sustainable healthy diet score is computed from 10 items, each of which is treated continuously from 0 to 1 point; for example, the scoring of red and processed meat consumption is as follows: 0 points for the consumption of >2 servings of red and processed meat and 0.25, 0.5, 0.75, and 1 points for the consumption of 1.5, 1, 0.5, and 0 servings per week, respectively. Food consumption will be assessed as the average daily food consumed during the entire assessment week. The scoring criteria and rationales are listed in Table 3. The total score of the environmentally sustainable healthy diet index will be the sum of the points for each item. Thus, the environmentally sustainable healthy diet index will range from 0 to 10 points, with 0 points being the lowest score in terms of environmentally sustainable healthy diet and 10 points being the highest (best) score (Table 3). Our overall scoring criteria are based on the recommendations of reference entities in the field of nutrition, mainly the EAT-Lancet Commission, Food and Agriculture Organization, and World Health Organization [25,40,41].

**Table 3:** Environmentally sustainable healthy diet index: scoring criteria.

Item	What does it assess?	Scoring criteria^a^	Rationale
Red and processed meat	Average daily red and processed meat consumption	1 point: 0 serv/w^b^ (or <0.15 serv/d^c^) 0 point: >2 serv/w (or >0.29 serv/d)	Ideally, no red or processed meat should be consumed in a sustainable healthy diet; however, up to 2 servings of red meat per week is acceptable [25].
Dairy products	Average daily dairy product consumption	1 point: ≤1 serv/d 0 point: >2 serv/d	In a sustainable healthy diet, the optimal intake of dairy products should be at the lower end of the range 0 to 500 mL (milk equivalents) [25].
Legumes and soy	Average daily legume and soy product consumption	1 point: ≥1 serv/d 0 point: 0 serv/d	Plant-based proteins should be the main protein source in someone’s diet, for their own health and for that of the planet. At least 1 serving of legumes per day should be consumed in a sustainable healthy diet [25].
Fruits and vegetables	Average daily fruit and vegetable consumption	1 point: ≥5 serv/d 0 points: <1 serv/d	At least 5 servings of fruits and vegetable (in total) should be consumed per day [25]. Less than 1 serving per day will be scored as 0 points owing to their crucial daily consumption for a healthy diet.
Nuts (including peanuts)	Average daily nut consumption	1 point: 1-2 serv/d 0 point: 0 serv/d and ≥4 serv/d	Daily consumption of nuts is recommended owing to their health benefits. Indeed, they have been suggested even as a replacement for red meat. However, the quantity should be moderate, as some nut-tree crops are water, fertilizer, and pesticide intensive [15,25].
Sugary, salty, and ultraprocessed foods	Average daily sugary, salty, and ultraprocessed food consumption	1 point: 0 serv/w 0 point: >2 serv/w (or >0.29 serv/d)	These foods should be consumed as less as possible; ideally, they should be avoided or at least consumed no more than once a week [40].
Whole grains	Proportion of servings of whole grains in relation to the total servings of all grains	1 point: 100% whole grains 0 point: 0% whole grains	Whole grains should be prioritized over their refined versions [25].
Oils and fats	Proportion of servings of virgin or extra virgin olive oil in relation to the total added fat intake	1 point: 100% virgin or extra virgin olive oil 0 point: 0% virgin or extra virgin olive oil	Unsaturated and nonrefined oils, mainly virgin olive oil (extra or not) in our context, should be prioritized over other dietary fats [25].
Alcoholic drinks	Average daily alcoholic drink consumption	1 point: 0 serv/w 0 point: >2 serv/w (or >0.29 serv/d)	Ideally, no alcohol should be consumed. According to the World Health Organization, the impact of alcohol consumption on chronic and acute health outcomes is largely determined by the total volume of alcohol consumed [41].
Other beverages: sodas, juices, energy drinks, etc	Average daily consumption of other unhealthy drinks	1 point: 0 serv/w 0 point: >2 serv/w (or >0.29 serv/d)	These foods should be consumed as less as possible; ideally, they should be avoided or at least consumed no more than once a week [40].

^a^Scores will range proportionally from 0 to 1 points. For instance, 0 points will be assigned for the consumption of >2 servings of red and processed meat per week and 1 point for 0 servings per week; 0.25, 0.5, and 0.75 points will be assigned for the consumption of 1.5, 1, and 0.5 servings per week, respectively.

^b^serv/w=servings per week.

^c^serv/d=servings per day.

##### Mechanisms of Action

Seven items capturing key psychological or motivational mechanisms of action that could potentially play a role in the adherence to sustainable healthy diets will be measured once during each assessment week (refer to objective 2.1 in the *Introduction* and *Statistics* sections). All items are presented in Table 4. Motives for purchasing or eating foods related to dietary sustainability and healthiness (ie, health, environment, affordability, and ethical) will be captured with 4 items adapted from eating motivation validated scales [42,43]. Another item, selected from a validated 12-item questionnaire, will be used to evaluate ecoanxiety, a construct that has been previously described as a relevant mediator of proenvironmental behavior changes [44]. Habit strength of purchasing and eating food while thinking about sustainability will be measured using an item adapted from the Self-Report Habit Index and Self-Report Behavioral Automaticity Index [45,46], and finally, self-efficacy toward following a sustainable healthy diet will be assessed using another item [47]. The comprehension of these questions was tested by following the same methodology as the one stated earlier for text message content.

**Table 4:** Items for food-related motives, ecoanxiety, habits, and self-efficacy.

Category and name			Item		Answer options
**Motives**					
	Health	In the last 7 days, how important was the healthiness of your food when choosing what to eat?		VAS^a^ 0-10: 0=not important at all and 10=very important	
	Environmental impact	In the last 7 days, how important was the environmental impact of your food (ie, carbon footprint, water footprint, etc) when choosing what to eat?		VAS 0-10: 0=not important at all and 10=very important	
	Fair and ethical sources	In the last 7 days, how important was the working conditions of the producers or sellers of your food when choosing what to eat or where to buy?		VAS 0-10: 0=not important at all and 10=very important	
	Price	In the last 7 days, how important was the price of your food when choosing what to eat?		VAS 0-10: 0=not important at all and 10=very important	
**Ecoanxiety**					
	Ecoanxiety	Over the last week, how often have you felt nervous, anxious or on edge, when thinking about climate change?		VAS 0-10: 0=never and 10=almost everyday	
**Habit strength**					
	Habit strength	Selecting healthy foods with low environmental impact and from fair sources is something that I do automatically, without thinking about it		VAS 0-10: 0=totally in disagreement and 10=totally in agreement	
**Self-efficacy**					
	Self-efficacy	To what extent do you feel capable of following a sustainable healthy diet?		VAS 0-10: 0=not at all confident and 10=totally confident	

^a^VAS: visual analog scale.

#### Relevance of the Interventional Text Messages

Finally, when sending motivational and educational messages as part of the intervention, we will ask the participants to indicate whether the message is “useful, informative or motivating” or “not useful, not informative or not motivating” in a visual analog scale. This will help us refine our messages for future studies and also control for engagement with the messages, given that an absence of answers will indicate that the participants did not read the message.

#### Qualitative Measurements

We will conduct 3 semistructured qualitative interviews to gain a deeper understanding of the intervention’s effect as well as to assess the feasibility, delivery, and acceptability of the intervention beyond the collected quantitative information. We anticipate that the qualitative data will allow us to complement the quantitative data in a way that adequately captures the contextual, social, and cultural factors that determine the intervention’s efficacy and perceived influence.

The first interview will be performed at baseline (during the first 2 weeks; refer to Figure 1) to investigate the eating behaviors of the participants (ie, habitual diet; favorite foods; sources of food; frequency of eating out; and the cultural, social, and personal backgrounds that may determine their behaviors), explore aspects related to sustainable healthy diets (ie, conceptualization of a sustainable healthy diet, participants’ views on what should change in their daily lives to achieve such a diet, previous attempts to improve their diet, and measures implemented in those attempts), and explore participants’ expectations of the intervention and their motivation for participating in the study. The second interview will be held at the end of the intervention (month 7) to examine participants’ perceptions of the effectiveness of the intervention (ie, changes in their eating habits and behaviors and other changes beyond diet, such as variations in their well-being and weight) as well as potential mediators that may have influenced outcomes that were not observable in the quantitative part; determine whether the intervention has been feasible (ie, any difficulties with the app, problems completing the questionnaires, understandability of the text messages, etc); identify potential improvements to the intervention’s delivery; and examine participants’ views on its acceptability, which includes engagement with both the evaluations and intervention’s features and any other relevant practical aspects of the study that the participants would like to discuss. The third interview will be conducted at the end of the follow-up period (month 12) and will be less structured than the previous ones, depending on the outcomes for each participant. The objective of the third interview will be to evaluate the persistence of the acquired eating behaviors, focusing on the personal, social, and contextual facilitators and barriers that may influence the persistence of the outcomes over time, as well as the role of the intervention in this persistence. The interview guides are included in Multimedia Appendix 6.

The objectives and procedures of the interviews will be explained at the beginning of the study in the face-to-face meeting for installing the app, in which each participant will confirm their willingness to participate. The researcher who will conduct the interviews will contact them to schedule an interview at each of the 3 time periods. The same researcher will be in charge of all the interviews. The interviews will be conducted on the web using the preferred platform of the participants (Google Meet [Google LLC], Zoom [Zoom Video Communications Inc], Signal [Signal Messenger LLC], WhatsApp [Meta Platforms Inc], etc), will last between 45 minutes and 1 hour, and will be audio recorded. During the interviews, prompts and probes will be used to collect rich and comprehensive data. All interviews will be digitally recorded, anonymized, and transcribed verbatim. Audio files and transcripts will be imported into the NVivo software (QSR International), which will be used for the data analysis.

### Data Analyses

#### Sample Size and Power Analyses for the Quantitative Side of the Project

The sample size for this pilot study is constrained by financial and human resources. We plan to recruit a total of 21 participants, with 7 participants in each subjective socioeconomic group (refer to the *Participants* section). However, in terms of statistical power, we will benefit from the intensive longitudinal nature of the study, that is, 15 evaluation weeks and up to 105 daily observations per participant. For objective 1.1.a, for which a weekly environmentally sustainable healthy diet index will be used, up to 315 observations will be available in total (21 participants × 15 evaluation weeks) and 105 observations per socioeconomic group (7 participants × 15 evaluation weeks). For objective 1.1.b, for which the daily raw data will be used, up to 2205 observations will be available in total (21 participants × 105 daily assessments), 735 observations in the 3 socioeconomic groups (7 participants × 105 daily assessments), and 105 observations at the individual level (as a general rule of thumb, a minimum of 70 observations are required for conducting individual-level analyses [48]). Given the pilot nature of this trial, we conducted power simulation analyses only for objective 1.1.a (ie, change in the weekly level of food healthiness and environmental sustainability). Considering group-level analyses and the analytical strategy detailed in the next section and assuming an increase of 2 units in the environmentally sustainable healthy diet score per phase, simulated power analysis indicated an adequate power value of 96%. The simulation was performed by generating the response variable as follows:

- Fixed effects: the intercept was set at 6, and the fixed coefficient for the phase was set as −2 for the initial phase and +2 for the final phase based on our average expected change in the main outcome.
- Random effects: it was assumed that each participant would have both a random slope and a random intercept and that both followed normal distributions, with SDs of 2 for the intercept and 0.5 for the slope. The correlation between them was set as 0.2. The random effect for each participant was created by drawing random numbers from a multivariate normal distribution whose covariance matrix was specified using these parameters (R mvrnorm from the package *MASS*).

Then, a linear mixed regression was performed on the data (R lme from the package *nlme*) using phase as a fixed effect and specifying a random slope and intercept for each participant across time. This process was repeated 1000 times, and the significance level for the fixed effect was 0.05.

#### Quantitative Data Analysis

Analyses for this project will be conducted with the statistical software R (R Foundation for Statistical Computing) [49]. The code and data generated for this study will be made open access when available.

##### Objective 1.1.a: Changes in the Environmental Sustainability and Healthiness of Diet

To test the main effect of the intervention on the environmental sustainability and healthiness of diet at the group level, linear mixed regression will be performed and expressed as measurement occasions (here, going from 1, first evaluation week, to 15, last evaluation week), using an ordered 3-categories phase variable as a fixed effect, which takes the values of baseline, intervention, and follow-up. Polynomial functions will also be fitted to the time variable to determine whether the index demonstrates any nonlinear patterns across time. If nonlinear patterns are detected, then other patterns (eg, quadratic, cubic, and piecewise) will be examined and selected based on visual inspections and goodness-of-fit indicators. Moderation analysis will be performed by exploring the role of the subjective socioeconomic level (ie, 3 socioeconomic groups entered as fixed effect) with regard to changes in diet. Fixed baseline characteristics will be included as control variables (ie, age, gender, education, income, and political orientation). Individual trajectories will be plotted, and heterogeneity in participants’ responses to the intervention will be discussed based on visual analyses using relevant guidelines for n-of-1 trials [50] (individual weekly changes in the whole diet will not be statistically modeled because only 15 observations will be available per participant).

##### Objective 1.1.b: Changes in Individual Food Groups and Food Waste

An analytical strategy similar to that used for objective 1.1.a will be followed for testing the effect of the intervention on specific food groups, including red and processed meat, fruits and vegetables, ultraprocessed foods, and dairy products, and food waste. A linear mixed model similar to that performed for the first objective will be performed, but time will be expressed as measurement days instead of weeks, thus ranging from 1 to 105. In addition to the group and subgroup analyses performed for objective 1.1.a and maximizing the higher number of observations per participant within those outcomes, individual-level models will also be used to quantify the effect of the intervention on each participant separately from the others by following an idiographic approach.

##### Objective 1.2: Feasibility of the Intervention

This objective will mainly be investigated through the qualitative data analyses reported in the next section. Simple quantitative analyses will be conducted to examine the relevance of our motivational and educational messages (ie, scores provided to the text messages at the individual and collective levels). We also report the percentage of missing values for each measurement burst.

##### Objective 2.1: Identifying Potential Mechanisms of Action Mediating the Effect of the Intervention on Eating Behaviors

Longitudinal mediation analyses will be conducted to understand the processes through which the intervention will potentially affect the environmental sustainability of eating behaviors. These analyses will be conducted independently for the 7 potential mediators presented in the section *Mechanisms of Action* (ie, motives for health, environmental, social, and affordability; ecoanxiety; habits; and self-efficacy) and by following recent guidelines based on longitudinal multilevel structural equation models [51]. These analyses will be performed for the total sample and per socioeconomic subgroup with the environmentally sustainable healthy diet index as the first dependent variable and, in secondary analyses, with the weekly level of the relevant food outcomes mentioned in objective 1.1.b (ie, red and processed meat, fruits and vegetables, ultraprocessed food, dairy products, and food waste). These food groups and food waste will be analyzed here at the weekly level to match the temporal resolution of the mechanisms of action that are also assessed weekly (and not daily).

##### Objective 2.2: Identifying Potential Covariations and Spillover Effects Among Food Groups and Between Food Composition and Food Waste

Covariations and spillover effects will be examined using network visualizations of the partial correlation matrix by following published guidelines and previous applications in the health behavior domain [52,53]. The within-participant network will be computed by subtracting the mean of each participant’s behavior across the 15 assessment weeks from their scores at each time, thus estimating a network of interrelationships centered on each person’s “average” behavioral pattern over 12 months. This method allowed us to test whether variations from the mean level for one behavior are associated with variations from the mean for other behaviors and, hence, covariation or spillovers. These networks will be estimated for the total group and the 3 socioeconomic subgroups using the 12 items presented in Table 2. Data from the 3 phases will be combined together to maximize the statistical power.

##### Objective 2.3: Potential Interaction of Socioeconomic Status With the Intervention Effectiveness

As mentioned in the previous sections, the role of socioeconomic status will be explored in objectives 1.1.a, 1.1.b, 2.1, and 2.2 by conducting group-specific analyses for the low, moderate, and high socioeconomic groups (refer to the above-mentioned sections for further details).

#### Qualitative Data Analysis

The qualitative data will be analyzed using framework analysis, a thematic-oriented approach to qualitative data analysis that is gaining popularity in the health sciences and is well suited for analyzing data from semistructured interviews [54,55]. The output of this method is a matrix containing the participants in the rows, analytical codes in the columns, and a data summary in the cells. Using this matrix, researchers can compare the participants’ views within each participant and across participants, thereby identifying patterns across the data set (ie, code-based analysis) while preserving the context of the participants’ views (ie, analysis by case). In addition, because of the structured nature of the method, framework analysis is helpful in studies that involve multidisciplinary research teams with a majority of members having quantitative expertise, such as this study. In this study, framework analysis will be implemented according to the following 6 steps outlined by Gale et al [55]: familiarizing with the interview transcripts, coding the transcripts, developing a working analytical framework by grouping the codes, applying the analytical framework to the transcripts, charting data into the framework matrix, and interpreting the data. Two researchers will independently code and apply the analytical framework. Disagreements between the researchers will be discussed until a consensus is established, if required, with the assistance of a third researcher.

#### Integration of the Quantitative and Qualitative Data

After completing all the analyses, a back-and-forth process will be used to integrate the quantitative and semistructured interview findings [56]. Joint displays will be used to facilitate this process, an increasingly used visual tool designed for facilitating integration in mixed methods studies [57]. Specifically, using joint displays, both quantitative and qualitative findings for objectives 1.1.a, 1.1.b, 1.2, and 2.1 will be juxtaposed, and the extent to which the qualitative results confirm, reject, complement, or expand on the quantitative results will be assessed. By integrating the quantitative and qualitative findings, a more comprehensive and fine-grained understanding of the effectiveness, feasibility, and implementation of the intervention will be gained.

### Ethics Approval

This study was approved by the Drug Research Ethical Committee (CEIm) of Parc de Salut MAR on October 19, 2022 (2022/10304/I), and will be conducted in accordance with the Helsinki Declaration. The participants will be required to provide written informed consent to participate in the study during the baseline visit before any study procedure.

## Results

This study was approved by the Ethics Committee on 19 October, 2022. It started in October 2022. The initial results are expected to be published in May 2023. The final results are expected by October 2023.

## Discussion

### Principal Findings

With regard to objective 1.1.a, we expect environmentally sustainable healthy diet to positively change over time (increasing score for the environmentally sustainable healthy diet index) with small to medium effect sizes, as observed in most previous behavior change interventions [14]. With regard to objective 1.1.b, which focuses on food groups and food waste and reflects the changes in the index, we expect the consumption of meat, dairy products, and ultraprocessed food to decrease over time and the consumption of unprocessed or minimally processed plant-based foods to increase. However, the rate of change might differ from one food group to another; for example, an increase in fruit and vegetable consumption might occur earlier in the intervention than a decrease in meat consumption, which might indicate that this second behavior is more difficult to change. At the individual level, we also expect the shape of those changes to be heterogeneous across participants, with linear improvements, 1-step changes, and inverse U–shape, for example. Objective 1.2 (feasibility of the study) is not hypothesis driven.

With regard to objective 2.1, we expect substantial mediation effects of motivations, ecoanxiety, habit, and self-efficacy on eating behaviors, with positive changes in eating behaviors being partially explained by positive changes in these variables. More specific hypotheses can also be formulated; for example, we expect motivation for protecting the environment to mediate changes in the food groups with the highest environmental impact (eg, meat) more than those in the food groups mainly relevant for health (eg, ultraprocessed food).

We do not have clear and specific hypotheses regarding the covariations among food groups and among dietary outcomes (objective 2.2) and hypotheses regarding the moderating role of participants’ socioeconomic level (objective 2.3), given that only a few studies have examined these dimensions before; overall, the participants from the lowest socioeconomic group may present a less healthy and environmentally sustainable diet than the participants belonging to the higher socioeconomic groups [27,58].

### Limitations

Our primary outcome is a weekly score of environmentally sustainable healthy diet created based on daily food measures to avoid recall bias. This specific questionnaire and scoring procedure have not yet received proper validation. However, this does not constitute a limitation to us. The questionnaire gathered common food groups and framed them as in other digital food frequency questionnaires validated for the Spanish population [59]. It provides information about specific foods gathered in each group with the serving size. The reason for using this customized tool instead of any previously validated is that this study is among the first to monitor changes in environmentally sustainable healthy diet as a whole and through dynamic daily assessments (instead of lower resolution measures, eg, monthly assessment), thus justifying the use of a new and shorter measurement tool. Similarly, the lack of validation of our scoring procedure is not a major limitation given that (1) this study is a pilot study and the data gathered will also be used to refine our scoring procedure later in time and that (2) the score is developed in a straightforward manner based on the EAT-Lancet Commission, and Food and Agriculture Organization, and World Health Organization recommendations [25,40,41]. Our team is conducting parallel validation studies of this questionnaire and scoring procedure, and we also expect to use the data from this pilot intervention to examine the sensitivity of the tool to detect behavior changes (with the ultimate aim of refining this questionnaire and scoring procedure for future interventions).

### Comparison With Prior Work

We prefer performing n-of-1 trials rather than adopting other traditional designs, such as parallel randomized control trials, because the former present some advantages, including (1) the possibility of developing efficient studies (in terms of time and human and financial resources) with few participants involved but providing forms of causal inference to quantify the intervention’s effectiveness for each participant (ie, within-individual randomization and time effect [60]); (2) the possibility of optimizing and refining the intervention over the course of the study based on the empirical data collected from the participants’ feedback [61]; and (3) the possibility of exploring heterogeneity between participants given that statistical inference can be produced at the individual level, which makes the tailoring of current and subsequent personalized interventions easier [62].

In recent years, some studies have been designed or implemented with the aim of promoting sustainable healthy eating behaviors [12,13]. However, to the best of our knowledge, this is the first study to address the overall dietary pattern, food waste, and socioeconomic dimensions of food in an intervention. In addition, the brief and continuous assessment implemented through the app will provide, for the first time in the domain of sustainable healthy diet, a high-resolution view of behavior changes useful for improving our understanding of the overall process compared with studies using traditional low-resolution measurement paradigms (eg, baseline and 3- and 6-month follow-ups). If we are able to manage potential technological issues that could happen with the app, the use of technology to monitor participants remotely should also help make this intervention minimally burdensome for the participants, as they do not need to attend in-person meetings.

### Conclusions

This study will address the urgent topic of adopting overall sustainable healthy diets, thus filling a gap in the literature and providing pilot data for scaling up the intervention in a full randomized controlled trial. It will provide insights into the effectiveness of the pilot intervention in changing eating behaviors toward a sustainable healthy diet, considering the whole dietary pattern and, hence, spillovers among food groups and among dietary outcomes; it will help to determine the moderation and interaction effects of several individual characteristics, such as socioeconomic level and gender; and it will address its own feasibility to determine the barriers to and enhancers of the intervention, which will help us refine and optimize the intervention for future studies.

## Appendix

Multimedia Appendix 1 Google form used for checking the inclusion and exclusion criteria (English and Spanish versions).

Multimedia Appendix 2 Scoring criteria for the dietary questionnaire included in the Google form to select participants.

Multimedia Appendix 3 Text messages (English and Spanish versions) to be sent to the participants during the intervention phase along with the behavior change techniques they match.

Multimedia Appendix 4 Baseline questionnaire (English and Spanish versions).

Multimedia Appendix 5 Supportive material showing specific food items gathered in the assessed food groups and examples of 1 and 0.5 servings (English and Spanish versions).

Multimedia Appendix 6 Interview guide (English and Spanish versions).

## Abbreviations

SCRIBE: Single-Case Reporting Guideline In Behavioral Interventions

## Data Availability

The data sets generated during or analyzed during this study will be available at Open Science Framework [63].
