# Supplementary material for: A Smartphone Intervention to Promote a Sustainable Healthy Diet: Protocol for a Pilot Study
Source: JMIR Res Protoc. 2023 Mar 2;12:e41443. doi: 10.2196/41443 (PMC10020902; doi:10.2196/41443)
Supplement: Multimedia Appendix 1 [file resprot_v12i1e41443_app1.pdf]

Supplemental material 1: Google form used for checking inclusion and exclusion criteria (English and Spanish versions)

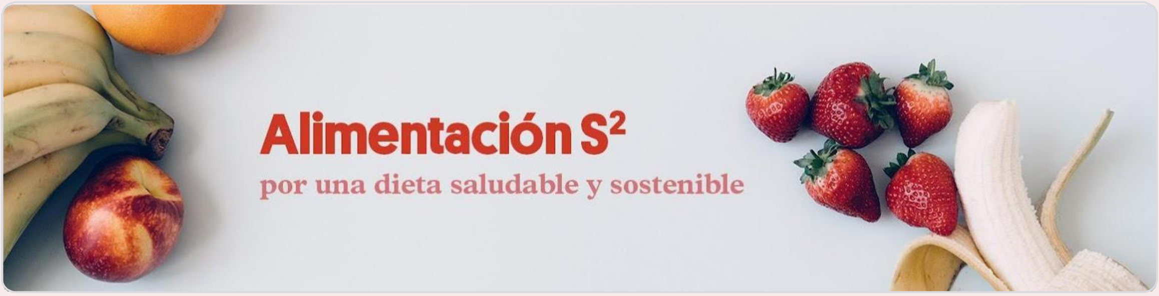

Alimentación S2: por una dieta saludable y sostenible

First of all, thank you very much for your interest!

We are looking for participants for our study, which aims to evaluate the effectiveness of an intervention that promotes sustainable healthy diets. To check if you meet the inclusion criteria and can be part of it, please fill in the following questionnaire.

ujue.fresan@isglobal.org (no compartidos) Cambiar de cuenta

*Obligatorio

How old are you? *

Tu respuesta

Do you have a mobile phone that allows you to install apps? *

- ○ Yes
- ○ No

Do you speak and read Spanish fluently? *

- ○ Yes
- ○ No

Where do you live? (Name of your city/town) *

Tu respuesta

On a scale from 1 to 10, where 1 represents people with less purchasing power, less education, with a worse job or no job , and 10 represents people with more purchasing power, higher level of education and better work, How would you rank yourself in relation to the inhabitants of Barcelona? *

	1	2	3	4	5	6	7	8	9	10	
Less purchasing power, less education, with a worse job or no job	◯	◯	◯	◯	◯	◯	◯	◯	◯	◯	Greater purchasing power, higher level of education and better work

If you are a woman, are you in any of these situations? i. You are pregnant ii. You plan to become pregnant during the next year iii. You have given birth less than 3 months ago iv. Your are breastfeeding. *

- ◯ Yes
- ◯ No
- ◯ Maybe
- ◯ I am not a woman

Are you a professional athlet? *

- ◯ Yes
- ◯ No

Have you ever been diagnosed with any food allergy/intolerance or other food-related pathology (e.g., celiac disease, non-celiac gluten sensitivity, nut allergy, etc.? *

- ◯ Yes
- ◯ No

Have you ever been diagnosed with any chronic diseases, such as cancer, diabetes, cardiovascular disease, obesity, etc.? *

- Yes
- No

Have you ever been diagnosed with any eating disorder (e.g., anorexia nervosa, bulimia nervosa, binge eating disorder, etc.) or other psychological/psychiatric condition (depression, anxiety, schizophrenia, bipolar disorder, etc.)? *

- Yes
- No

Are you following any special restrictive dietary pattern? *

- No
- Yes, gluten free
- Yes, low in carbohydrates or sugars
- Yes, sliming diet
- Other

Do you make your own food decisions? *

- Yes, I choose what I eat most of the time
- No, someone else does it for me (for example, your husband/wife/partner, mother/father decides most meals for you)

Siguiente

Borrar formulario

Nunca envíes contraseñas a través de Formularios de Google.

Este formulario se creó en ISGlobal. Notificar uso inadecuado

Google Formularios

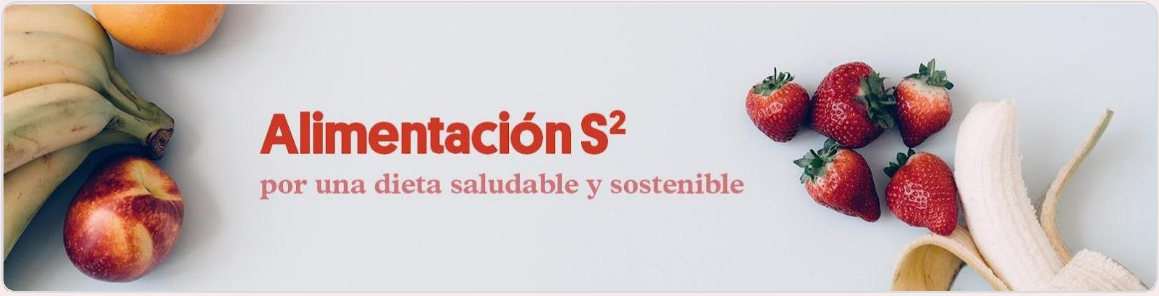

Alimentación S2: por una dieta saludable y sostenible

ujue.fresan@isglobal.org (no compartidos) Cambiar de cuenta

*Obligatorio

Regular diet in the last month

In this section we will ask you some questions to know how your eating behaviors during the last month. The goal is not to judge you, but to know your usual eating behaviors, to evaluate if our study can help you improve them, if necessary. So please try to reponse as close to reality as possible.

How often did you eat the following food groups during the last month? *

	Never	1-3 times per month	1 time per week	2-5 times per week	1 time per day o almost 1 time per day	2 times per day	3-4 times per day	5 o more times per day
Red and/or processed meat: beef, pork and lamb, offals (liver, kidney, etc.)	○	○	○	○	○	○	○	○
Dairiy products: milk, yogurt, cheese	○	○	○	○	○	○	○	○
Legumes: any type (chickpeas,, lentils, beans, etc.) and soy derivatives such as tofu or texturized soy.	○	○	○	○	○	○	○	○
Fruits and/orr vegetables	○	○	○	○	○	○	○	○
Nuts and seeds: any kind (nuts,, pistachios, pumpkiin seeds, chia seeds, etc.) toasted or natural, and their butters withoutadded	○	○	○	○	○	○	○	○

sugar (peanut butter, tahini, etc.)								
Hiighlly–processed foods,, rich in sugars, salt and/or fats, such as biscuits, pastries, chips and other snacks, desserts such as custards, etc.	○	○	○	○	○	○	○	○
Alcoholic drinks: any type, including wine and beer	○	○	○	○	○	○	○	○
Sodas, juices, energy drniks	○	○	○	○	○	○	○	○

Of all the cereal-based foods (bread, pasta, rice) you have eaten in the last month, what proportion has been whole grain? *

	0	1	2	3	4	5	6	7	8	9	10	
Not taken any whole grain foods	○	○	○	○	○	○	○	○	○	○	○	All the cereal-based foods I have taken have been whole grain

Of all the oils and fats (olive, sunflower, corn, margarine, butter, etc.) you have used in the last month, what proportion has been of virgin or extra virgin olive oil? *

	0	1	2	3	4	5	6	7	8	9	10	
I have not used virgin or extra virgin olive oil	○	○	○	○	○	○	○	○	○	○	○	All the oil I used was virgin olive or extra virgin

Atrás Siguiente Borrar formulario

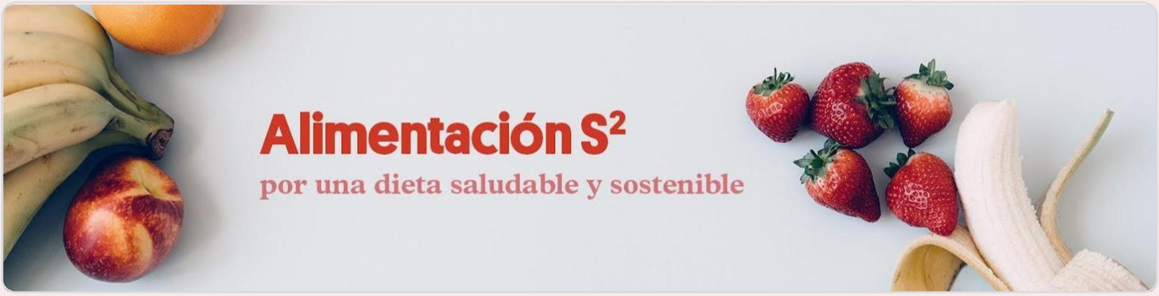

Alimentación S2: por una dieta saludable y sostenible

ujue.fresan@isglobal.org (no compartidos) Cambiar de cuenta

*Obligatorio

Thank you very much for participating

Please enter your email address. We will only use it to let you know if you have been selected to participate in the study. *

Tu respuesta

Atrás Enviar Borrar formulario

Nunca envíes contraseñas a través de Formularios de Google.

Este formulario se creó en ISGlobal. Notificar uso inadecuado

Google Formularios

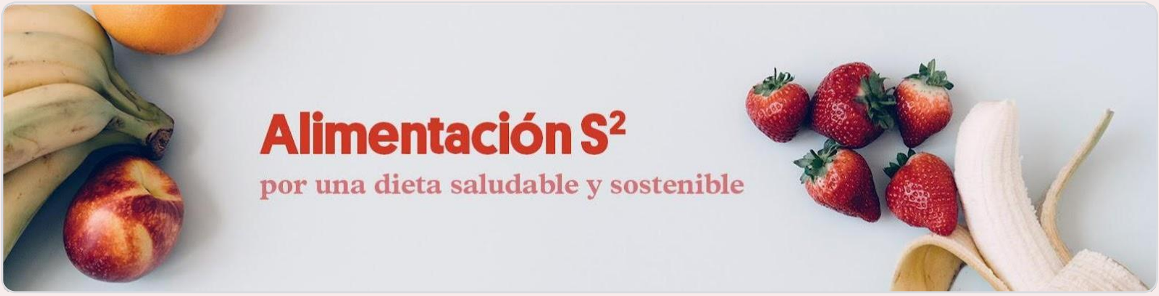

Alimentación S2: por una dieta saludable y sostenible

En primer lugar, ¡muchas gracias por su interés!

Estamos buscando participantes para nuestro estudio, cuyo objetivo es evaluar la eficacia de una intervención en la que se promueven dietas saludables y sostenibles. Para comprobar si usted cumple los requisitos y puede formar parte del mismo, por favor, rellene el siguiente cuestionario.

ujue.fresan@isglobal.org (no compartidos) Cambiar de cuenta

*Obligatorio

¿Cuántos años tiene? *

Tu respuesta

¿Tiene un teléfono móvil que permite instalar aplicaciones (apps)? *

- Sí
- No

¿Habla y lee español de manera fluida? *

- Sí
- No

¿En qué pueblo/ciudad reside? *

Tu respuesta

En una escala del 1 al 10, donde el 1 representa las personas con menor poder adquisito, menos estudios, con peor trabajo o sin trabajo, y el 10 las personas con mayor poder adquisito, mayor nivel de estudios y mejor trabajo, ¿en qué número se posicionaría usted respecto a los habitantes de Barcelona? *

	1	2	3	4	5	6	7	8	9	10	
Menor poder adquisito, menos estudios, con peor trabajo o sin trabajo	○	○	○	○	○	○	○	○	○	○	Mayor poder adquisito, mayor nivel de estudios y mejor trabajo

Si es mujer, ¿se encuentra en alguna de estas situaciones? i. Está embarazada ii. Planifica quedarse embarazada durante el próximo año iii. Ha dado a luz hace menos de 3 meses iv. Está dando el pecho. *

- ○ Sí
- ○ No
- ○ Tal vez
- ○ No soy mujer

¿Es deportista de manera profesional? *

- ○ Sí
- ○ No

¿Ha sido diagnosticado/a de alguna alergia/intolerancia alimentaria u otra patología relacionada con la alimentación (por ejemplo, celiaquía, sensibilidad al gluten no celíaca, alergia a frutos secos, etc.? *

- ○ Sí
- ○ No

¿Ha sido diagnosticado/a de alguna enfermedad crónica, como cáncer, diabetes, enfermedad cardiovascular, obesidad, etc.? *

- Sí
- No

¿Ha sido diagnosticado/a de algún trastorno de la conducta alimentaria (por ejemplo, anorexia nerviosa, bulimia nerviosa, trastorno por atracón, etc.) u otra afección psicológica/psiquiátrica (depresión, ansiedad, esquizofrenia, trastorno bipolar, etc.)? *

- Sí
- No

¿Sigue algún patrón dietético restrictivo especial? *

- No
- Sí, libre de gluten
- Sí, baja en carbohidratos/azúcares
- Sí, dieta de adelgazamiento
- Otra

¿Toma sus propias decisiones alimentarias? *

- Sí, elijo lo que como la gran mayoría de las veces
- No, otra persona lo hace por mí (por ejemplo, su marido/mujer/pareja, madre/padre decide la mayoría de las comidas por usted)

Siguiente

Borrar formulario

Nunca envíes contraseñas a través de Formularios de Google.

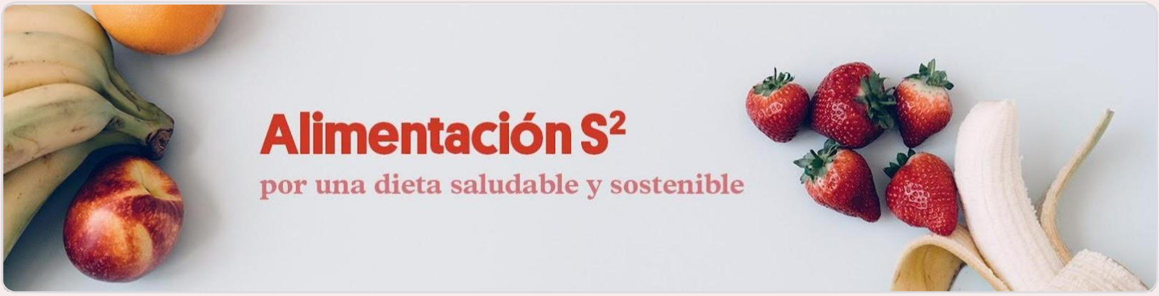

Alimentación S2: por una dieta saludable y sostenible

ujue.fresan@isglobal.org (no compartidos) Cambiar de cuenta

*Obligatorio

Dieta habitual en el último mes

En esta sección le vamos a hacer unas preguntas para conocer cómo se ha alimentado durante el último mes. El objetivo no es juzgarle, sino conocer su comportamiento alimentario habitual y ver si nuestro estudio puede ayudarle a mejorarlo en caso de ser necesario. Por ello, por favor, intente que sus respuestas sean los más ajustadas a a la realidad.

¿Con qué frecuencia consumió los siguientes grupos de alimentos durante el último mes? *

	Nunca	1-3 veces al mes	1 vez a la semana	2-5 veces a la semana	1 vez al día todos o prácticamente todos los días	2 veces al día	3-4 veces al día	5 o más veces al día
Carne roja y/o procesada: carne de ternera, cerdo y cordero, vísceras (hígado, riñón, etc.)	○	○	○	○	○	○	○	○
Lácteos: leche, yogur, queso	○	○	○	○	○	○	○	○
Legumbres: de cualquier tipo (garbanzos, lentejas, alubias, etc.) y derivados de la soja como tofu o soja texturizada	○	○	○	○	○	○	○	○
Frutas y verduras	○	○	○	○	○	○	○	○
Frutos secos y semillas: de cualquier tipo (nueces, pistachos, pipas de calabaza, semillas de chia, etc) tostados o naturales, y sus cremas sin azúcar añadido (crema de cacahuete, tahini, etc.)	○	○	○	○	○	○	○	○

Alimentos altamente procesados, ricos en azúcarres, sal y/o grrasa, como galletas, bollería, patatas fritas y otros snacks de bollsa, postres como natillas, etc.	○	○	○	○	○	○	○	○
Bebiidas alcohólicas: vino, cerveza, alcoholes, etc.	○	○	○	○	○	○	○	○
Refrrescos, zumos, bebiidas energéticas	○	○	○	○	○	○	○	○

De todos los alimentos a base de cereales (pan, pasta, arroz) que has comido en el último mes, ¿qué proporción ha sido integral? *

	0	1	2	3	4	5	6	7	8	9	10	
No tomado ningún alimento a base de cereales integrales	○	○	○	○	○	○	○	○	○	○	○	Todos los alimentos a base de cereales que he tomado han sido integrales

De todos los aceites y grasas (aceite de oliva, de girasol, de maíz, margarina, mantequilla, etc.) que has usado en el último mes, ¿qué proporción ha sido de aceite de oliva virgen o virgen extra? *

	0	1	2	3	4	5	6	7	8	9	10	
No he usado aceite de oliva virgen ni virgen extra	○	○	○	○	○	○	○	○	○	○	○	Todo el aceite que he usado ha sido de oliva virgen o virgen extra

Atrás Siguiente Borrar formulario

Nunca envíes contraseñas a través de Formularios de Google.

Este formulario se creó en ISGlobal. Notificar uso inadecuado

Google Formularios

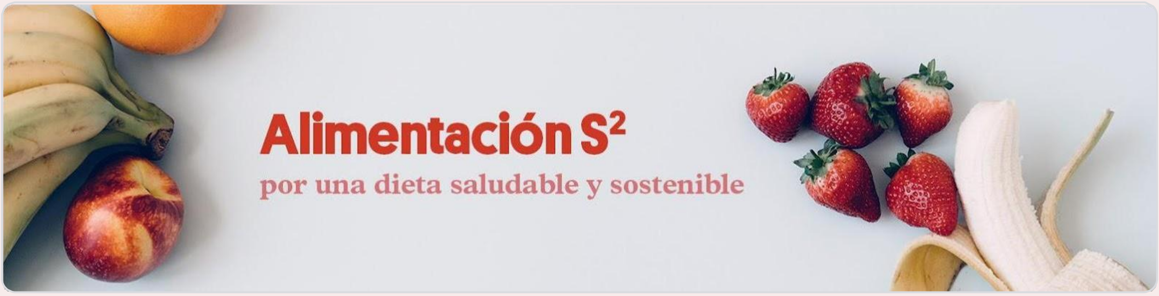

Alimentación S2: por una dieta saludable y sostenible

ujue.fresan@isglobal.org (no compartidos) Cambiar de cuenta

*Obligatorio

Muchas gracias por su participación

Por favor, indique su correo electrónico. Solo lo usaremos para ponernos en contacto con usted para comunicarle si ha sido seleccionado/a para participar en el estudio. *

Tu respuesta

Atrás Enviar Borrar formulario

Nunca envíes contraseñas a través de Formularios de Google.

Este formulario se creó en ISGlobal. Notificar uso inadecuado

Google Formularios
