# Supplementary material for: A Smartphone Intervention to Promote a Sustainable Healthy Diet: Protocol for a Pilot Study
Source: JMIR Res Protoc. 2023 Mar 2;12:e41443. doi: 10.2196/41443 (PMC10020902; doi:10.2196/41443)
Supplement: Multimedia Appendix 2 [file resprot_v12i1e41443_app2.pdf]

Supplemental material 2: Scoring criteria for the dietary questionnaire included in the Google Form to select participants.

FOOD GROUPS	NEVER	1-3 times/month	1 time/week	2-5 times/week	1 time/day	2 times/day	3-4 times/day	5 or more times/day
RED/PROCESSED MEAT	0	0	0	0.5	1	1	1	1
DAIRY PRODUCTS	0	0	0	0	0	0.5	1	1
LEGUMES	1	1	1	0.5	0	0	0	0
FRUITS AND VEGETABLES	1	1	1	1	1	0.5	0	0
NUTS AND SEED	1	1	0.5	0	0	0	0	0
ULTRPROCESSED FOODS	0	0	0	0.5	0.5	1	1	1
ALCOHOL	0	0	0	0.5	0.5	1	1	1
SOFT DRINKS, JUICES, ETC.	0	0	0	0.5	0.5	1	1	1
	**≤30%**		**40-60%**		**>60%**			
WHOLE GRAINS	1	1	0.5	0.5	0	0		
OILS	1	1	0.5	0.5	0	0		
