# Supplementary material for: A Smartphone Intervention to Promote a Sustainable Healthy Diet: Protocol for a Pilot Study
Source: JMIR Res Protoc. 2023 Mar 2;12:e41443. doi: 10.2196/41443 (PMC10020902; doi:10.2196/41443)
Supplement: Multimedia Appendix 3 [file resprot_v12i1e41443_app3.pdf]

Supplemental material 3: Text messages to be sent to the participants during the intervention phase along with the behavior change techniques they match (English and Spanish versions).

Date		Behavioral change technique	Content	
			English	Spanish
Month 1	Week 3	Welcome	You have already been part of the study "S2 eating: for a healthy and sustainable diet". THANK YOU! :) From now on and over the next few months, you will receive notifications with information and advices about healthy and sustainable diets. Let's start by explaining what a healthy and sustainable diet is. A healthy and sustainable diet is a dietary pattern that is not only good for your health, but also environmentally friendly, as well as fair and ethical with the people involved in its production and commercialization (farmers, collectors, distributors and retailers, etc.). Such a diet is characterized by being based on whole plant-based foods (legumes, whole grains, fruits, vegetables, nuts), with little or no food of animal origin (especially red meat, processed meat and dairy), in which food waste is minimal, and food is obtained from fair and ethical sources.<br>Know that, do you think that your diet is healthy and sustainable?	Ya llevas 2 semanas formando parte del estudio "Alimentación S2: por una dieta saludable y sostenible". ¡MUCHAS GRACIAS! :)<br><br>A partir de ahora y a lo largo de los próximos meses, irás recibiendo notificaciones con información y consejos sobre dietas saludables y sostenibles.<br>Comencemos por explicar qué es una dieta saludable y sostenible.<br>Una dieta saludable y sostenible es un patrón dietético que no solo es bueno para tu salud, sino también respetuoso con el medioambiente, así como justo y ético con las personas implicadas en su producción y comercialización (agricultores, recolectores, distribuidores y vendedores, etc.).<br>Una dieta tal se caracteriza por estar basada en alimentos de origen vegetal poco procesados (legumbres, cereales integrales, frutas, verduras, frutos secos), con poco o nada de alimentos de origen animal (especialmente carne roja, carne procesada y lácteos), en la que el desperdicio alimentario es mínimo, y los alimentos son obtenidos fuentes justas y éticas.<br>Sabiendo esto, ¿crees que tu alimentación es saludable y sostenible?

		2.3 Self-monitoring of behavior	Many times we are not fully aware of all the food and drinks we consume or how much we consume them. Keeping track of what you eat and drink can help us become aware of what our food is like. Try it for a few days! You might be surprised. You can make the registration on paper or using an electronic diary, which is most comfortable for you; try to write down what and how much you have eaten/drunk right after each intake, or as soon as possible, to avoid forgetting it.	Muchas veces no somos plenamente conscientes de todos los alimentos y bebidas que consumimos ni en qué cantidad los consumimos. Llevar un registro de lo que se come y se bebe nos puede ayudar a tomar consciencia de cómo es nuestra alimentación.<br>¡Pruébalo durante unos días! Quizás te sorprendas.<br>Puedes hacer el registro en papel o utilizando un diario electrónico, lo que te sea más cómodo; intenta anotar qué y cuánto has comido/bebido justo después de cada ingesta, o lo antes posible, para evitar posibles olvidos.
	Week 4	5.1 Information about health consequences	Did you know that poor nutrition causes more deaths worldwide than tobacco? We are adopting increasingly hypercaloric diets, high in meat and highly processed foods, rich in sugars, salt and fats, and with few plant-based foods, such as fruits, vegetables, whole grains and legumes. This dietary pattern is very unhealthy, and is generating the incidence of many diseases. In Spain, for example, 5.3 million people have type 2 diabetes, and half of the adult population is overweight or obese. Following a diet based on unprocessed plant-based foods-fruits, vegetables, legumes, nuts, and whole grains-is essential to enjoy good health.	¿Sabías que la mala alimentación genera más muertes a nivel mundial que el tabaco?<br><br>Estamos adoptando dietas cada vez más hipercalóricas, altas en carne y alimentos altamente procesados, ricas en azúcares, sal y grasas, y con pocos alimentos de origen vegetal, como frutas, verduras, cereales integrales y legumbres. Este patrón dietético es muy poco saludable, y está generando la aparición de muchas enfermedades. En España, por ejemplo, 5.3 millones de personas tienen diabetes tipo 2, y la mitad de la población adulta padece sobrepeso u obesidad.<br><br>Seguir una alimentación basada en alimentos de origen vegetal poco procesados -frutas, verduras, legumbres, frutos secos y cereales integrales- es fundamental para gozar de buena salud.

		5.4 Information about environmenta l consequences	Did you know that adopting a healthy and sustainable diet is one of the most effective strategies to fight climate change? Food production is one of the human activities that generate more greenhouse gases-those responsible for climate change. We consumers, reducing our consumption of meat and dairy products, and avoiding food waste, are a fundamental piece in the fight against the climate crisis.	¿Sabías que adoptar una dieta saludable y sostenible es una de las estrategias más eficaces para luchar contra el cambio climático? La producción de alimentos es una de las actividad humanas que más gases de efecto invernadero -los responsables del cambio climático- generan. Nosotros los consumidores, disminuyendo nuestro consumo de carne y lácteos, y evitando el desperdicio de comida, somos una pieza fundamental en la lucha contra la crisis climática.
Month 2	Week 5	2.3 Self-monitoring of behavior	A few days ago we invited you to keep a diary of your diet over a week to be aware of what you ate and how much you ate it. Now is time to reflect and respond to yourself: "Is my diet rich in unprocessed plant foods, such as fruits, vegetables, and legumes, or is it high in animal foods and products rich in sugars, salt, and fats?"	Hace unos días te invitamos a llevar un diario de tu alimentación a lo largo de una semana para ser consciente de qué comiste y en qué cantidad lo comiste. Ahora es momento de reflexionar y responderte a ti mismo: "¿Es mi dieta rica en alimentos vegetales poco procesados, como frutas, verduras y legumbres, o por el contrario es alta en alimentos de origen animal y productos ricos en azúcares, sal y grasas?"

		4.1 Instructions on how to perform a behavior	Main dietary changes to follow a healthy and sustainable diet<br>INCREASE: fruits and vegetables, legumes, nuts.<br>DECREASE: meats (especially red and processed), dairy and foods rich in sugar, salt and highly processed.<br>CHANGE TO: water as a drink of choice (instead of soft drinks, juices, alcoholic beverages), whole grains (instead of white), virgin olive oil (instead of sunflower, corn, butter...).<br>With these small changes in your diet you can follow a healthier and sustainable diet.<br>And you, are you willing/ to give them?<br>Here is an image that can serve as a reminder.	Principales cambios en la dieta para seguir una alimentación saludable y sostenible<br>AUMENTAR: frutas y hortalizas, legumbres, frutos secos.<br>DISMINUIR: carnes (sobre todo roja y procesada), lácteos y alimentos ricos en azúcar, sal y altamente procesados.<br>CAMBIAR A: agua como bebida de elección (en vez de refrescos, zumos, bebidas alcohólicas), cereales integrales (en vez de blancos), aceite de oliva virgen (en vez de girasol, maíz, mantequilla…).<br>Con estos pequeños cambios en tu dieta podrás seguir una alimentación más saludable y sostenible. Y tú, ¿estás dispuesto/a a darlos? A continuación te dejo una imagen que te puede servir como recordatorio.
	Week 6	5.4 Information about environmenta l consequences	Not all foods have the same environmental impact<br>In general, foods of animal origin are much more polluting and require many more resources than those of plant origin. Why? Let's have a look at the main reasons:<br>To eat food of animal origin, food to feed those animals had to be previously grown. And obviously, the production of their food requires resources and generates an impact on the environment. For example, to get 1 kilo of chicken, pork or cow, animals have to be fed more than 3 kg, 6 kg and 25 kg of food, respectively. Eating directly those food used for feed is more efficient to save resources and reduce the environmental impact of food.<br>On the other hand, manure derived from animal production, which generates gases with a global warming potential much higher than CO2 itself.<br>In addition, in the case of ruminants (cow, sheep and goat), their great contribution to climate change is not only due to these two factors, but also to the digestion process of these animals, in which they emit large amounts of greenhouse gases. In fact, beef,	No todos los alimentos tienen el mismo impacto ambiental<br>En general, los alimentos de origen animal son mucho más contaminantes y requieren muchos más recursos que los de origen vegetal. ¿Por qué? Veamos las principales razones:<br>Para comer alimentos de origen animal, previamente se habrá tenido que cultivar comida para alimentar a esos animales. Y, obviamente, la producción de su comida requiere recursos y genera un impacto en el medio. Por ejemplo, para obtener 1 kilo de pollo, cerdo o vaca, los animales tienen que ser alimentados con más de 3 kg, 6 kg y 25 kg de comida, respectivamente. Comer directamente nosotros esos alimentos es más eficiente para ahorrar recursos y reducir el impacto ambiental de la alimentación.<br>Por otro lado, el estiércol derivado de la producción animal, que genera unos gases con un potencial de calentamiento global muy superior al del propio CO2.<br>Además, en el caso de los rumiantes (vaca, oveja y cabra), su gran contribución al cambio climático no solo se debe a

			lamb and cheese are by far the foods with the greatest carbon footprint of our diet.<br>Hence the insistence on reducing the consumption of food of animal origin, especially meat and dairy, to follow a sustainable diet.	estos dos factores, sino también al proceso de digestión de estos animales, en el que van emitiendo grandes cantidades de gases con efecto invernadero. De hecho, la carne de ternera, la de cordero y el queso son, con diferencia, los alimentos con mayor impacto ambiental de nuestra dieta.<br>De ahí la insistencia en reducir el consumo de alimentos de origen animal, especialmente carne y lácteos, para llevar una dieta sostenible.
		5.1 Information about health consequences	Red meats, such as beef, lamb or pork, and processed meats, such as cold cuts, ham or sausages, have been classified as potentially carcinogenic by the Internationally Agency for Research on Cancer (IARC) and, in addition, can cause cardiovascular diseases, like stroke and heart attacks. For their impact on our health, and for their great environmental impact, a diet, to be healthy and sustainable, should not include more than one serving of red meat per week, and avoid processed meats.<br>And you, how much red and processed meat do you take? If it is more than recommended, would you be willing/ to reduce their consumption for your health and the planet?	Las carnes rojas -como ternera, cordero o cerdo- y las carnes procesadas -como los embutidos, jamón serrano o salchichas- han sido catalogadas como potencialmente cancerígenas por la Agencia Internacional de Investigación del Cáncer (IARC) y, además, pueden provocar enfermedades cardiovasculares, como ictus e infartos.<br>Por su repercusión en nuestra salud, y por su gran impacto ambiental, una dieta, para ser saludable y sostenible, no debe incluir más de una ración de carne roja a la semana, y evitar al máximo las carnes procesadas.<br>Y tú, ¿cuánta carne roja y procesada tomas? Si es más de lo recomendado, ¿estarías dispuesto/a a disminuir su consumo por tu salud y la del planeta?

	Week 7	5.1 & 5.4 Information about health and environmenta l consequences	Many people believe that meat is essential. But... reality is different.<br>Our body needs protein, not meat. Other foods such as fish, eggs, dairy products, legumes, and nuts are also sources of protein. Specifically, legumes-such as chickpeas, lentils or beans-are an excellent protein source. Several studies confirm that the consumption of legumes have many health benefits, such as reducing the risk of cardiovascular diseases, diabetes or obesity, among others. In addition, they are the protein food with the lowest environmental impact, much lower than that of dairy products, eggs, meat and fish. Therefore, prioritizing the consumption of legumes over protein foods of animal origin is essential to achieve a healthy diet with low impact.<br>In your diet, what is the main protein source? If it is legumes, congratulations!	Mucha gente cree que la carne es esencial. Pero... la realidad es otra.<br>Nuestro cuerpo necesita proteínas, no carne. Otros alimentos como el pescado, los huevos, lácteos, las legumbres y los frutos secos también son fuentes proteicas. En concreto, las legumbres -como los garbanzos, lentejas o alubias- son una excelente fuente proteica. Diversos estudios confirman que el consumo de legumbres reportan muchos beneficios para la salud, como reducir el riesgo de enfermedades cardiovasculares, diabetes u obesidad, entre otras. Además, son el alimento proteico con menor impacto ambiental, mucho menor que el de los lácteos, huevos, carne y pescados.<br>Por ello, priorizar el consumo de legumbres frente a alimentos proteicos de origen animal resulta imprescindible para conseguir una dieta saludable con bajo impacto ambiental.<br>En tu dieta, ¿cuál es la principal fuente proteica? Si son las legumbres, ¡enhorabuena!

		4.1 How to perform a behavior	If you want to change something in your diet, making a list with several alternatives could be useful. For example, if you try to reduce your consumption of red or processed meat, you can replace steaks with white meat, fish or legumes; prepare meatballs or burgers with legumes instead of with minced meat; make plant-based pâté sandwiches instead of with cold cuts; plant-based pasta sauces (e.g., pesto or arrabiata) instead of sauces with meat. Make a list, and include everything you can think of. You can also ask friends and family, maybe they can give you ideas you wouldn't even think of.	Si quieres cambiar algo de tu alimentación, es bueno hacer una lista con varias alternativas. Por ejemplo, si intentas reducir tu consumo de carne roja o procesada, puedes:<br>Reemplazar los filetes o el lomo por carne blanca, pescado o legumbres.<br>Preparar albóndigas o hamburguesas de legumbres en lugar de con carne picada.<br>Hacer bocadillos de patés vegetales en lugar de con embutido.<br>Salsas de base vegetal para la pasta (por ejemplo, pesto o arrabiata) en lugar de salsas con carne.<br>Haz una lista, e incluye todo lo que se te ocurra. También puedes preguntar a amigos y familiares, quizás te puedan dar ideas que ni se te hubieran ocurrido.
	Week 8	4.1 Instructions on how to perform a behavior	If you're not used to take legumes on a regular basis, your digestion can become heavy at first. To avoid this, here are 4 tips:<br>1. Start by eating crushed legumes (hummus, creams) and legumes of a small size, such as lentils.<br>2. Incorporate them into your diet gradually, starting with small portions that can be increased as your body gets used to<br>3. Leave them to soak overnight, and, if you can, change the soaking water at least once<br>4. Cook them during a long time, following the indications reported by the trademark	Si no estás acostumbrado/a a tomar legumbres de manera habitual, su digestión se te puede hacer pesada al principio. Para evitarlo, aquí tienes 4 consejos:<br>1. empieza comiendo legumbre triturada (hummus, cremas) y con legumbre de un tamaño pequeño, como las lentejas.<br>2. incorpóralas a tu dieta de manera progresiva, comenzando por raciones pequeñas que podrás ir aumentando conforme tu cuerpo se vaya acostumbrando<br>3. déjalas en remojo toda la noche, y, si puedes, cámbiales el agua de remojo al menos una vez<br>4. haz cocciones largas, siguiendo las indicaciones que reporta la marca comercial

		5.1 Information about health consequences	Many people avoid eating nuts because they think they will increase their weight. What a mistake! Although it is true that they contain many calories and should be consumed in moderation, the reality is that they are very satiating, so they help control the total calories taken throughout the day. In fact, people who eat a handful of nuts a day not only do not get fat, but they have better health. Numerous studies talk about the health benefits, especially for the heart, of taking a handful of nuts a day. The variety is immense: nuts, almonds, peanuts, pistachios, hazelnuts... But remember: to be healthy they must be natural or toasted, not fried or salted.<br>Do you usually eat a handful of nuts daily? If not, today would be a good moment to start.	Muchas personas evitan consumir frutos secos porque piensan que engordan. ¡Qué gran equivocación! Aunque es cierto que contienen muchas calorías y que se deben consumir con moderación, la realidad es que son muy saciantes, por lo que ayudan a controlar las calorías totales que se toman a lo largo del día.<br>De hecho, las personas que consumen un puñadito de frutos secos al día no solo es que no engorden, sino que tienen mejor salud. Numerosos estudios hablan de los beneficios para la salud, sobre todo para el corazón, de tomar un puñadito de frutos secos al día.<br>La variedad es inmensa: nueces, almendras, cacahuetes, pistachos, avellanas… Eso sí: para que sean saludables deben ser al natural o tostados, no fritos ni salados.<br>Y tú, ¿sueles tomar un puñadito frutos secos en tu día a día? Si no, quizás hoy pueda ser un buen momento para empezar

Month 3	Week 9	Recipe	If you have no idea how to fill your sandwich if it is not with cold cuts or cheese... How would you like to try a plant-based pâté sandwich? With this recipe you will incorporate legumes and nuts into your diet, while displacing unhealthy foods with a high environmental impact, such as cold cuts. You can find the recipe at the following link: https://drive.google.com/file/d/1E55NiyDiL1wbPgJ2n_KPuMCkVskJYkiv/view?usp=sharing	Si no tienes ni idea de qué hacerte el bocadillo si no es de embutido o queso... ¿Qué te parecería probar un bocadillo de paté vegetal? Con esta receta incorporarás legumbres y frutos secos a tu dieta, desplazando a su vez a alimentos no saludables y con un alto impacto ambiental, como los embutidos.<br>Encontrarás la receta en el siguiente link: https://drive.google.com/file/d/1E55NiyDiL1wbPgJ2n_KPuMCkVskJYkiv/view?usp=sharing
		5.1 Information about health consequences	Did you know that opting for whole grains is one of the best changes you can make in your diet to make it healthier? Opting for foods such as bread, pasta and rice in its whole version instead of white bread, pasta and rice brings numerous health benefits. Whole grains are naturally high in fiber, which helps us feel full, making it easier to maintain a healthy body weight. In addition, its use is associated with a lower risk of heart disease, diabetes, certain cancers and other health problems. We encourage you to give them an opportunity. If you are not used to taking them on a regular basis, you can incorporate them into your diet gradually without a radical change in your dietary pattern. For example, you can make your favorite pasta recipe	¿Sabías que optar por cereales integrales es uno de los mejores cambios que puedes hacer en tu dieta para que sea más saludable?<br>Optar por alimentos como pan, pasta y arroz en su versión integral en lugar de pan, pasta y arroz blancos aporta numerosos beneficios para la salud.<br>Los cereales integrales son naturalmente altos en fibra, lo que ayuda a que nos sintamos llenos, haciendo que sea más fácil mantener un peso corporal saludable. Además, su consumo se relaciona con un menor riesgo de enfermedades cardíacas, diabetes, ciertos cánceres y otros problemas de salud.<br>Te animamos a que les des una oportunidad.

			but using whole-grain pasta instead of white pasta. The sauce will make the change practically imperceptible.	Si no estás acostumbrado/a a tomarlos de manera habitual, puedes incorporarlos en tu dieta de manera progresiva sin que suponga un cambio radical de tu patrón dietético. Por ejemplo, puedes seguir haciendo tu receta de pasta favorita pero usando pasta integral en lugar de pasta blanca. La salsa hará que el cambio sea prácticamente imperceptible.
	Week 10	5.4 Information about environmental consequences 4.1 Instructions on how to perform a behavior	Did you know that 1 out of 3 foods produced are ultimately not consumed? Most of this waste is produced by us, consumers, in our homes. Saving leftovers to eat another time, keeping food well, or first consuming foods that have been at home longer to avoid spoiling are some of the strategies to reduce food waste. Below you will find a link if you want to discover different ways to reduce food waste from your own home. How many of them are you going to implement? https://www.eufic.org/es/seguridad-alimentaria/articulo/consejos-para-reducir-el-desperdicio-de-alimentos/	¿Sabías que 1 de cada 3 alimentos producidos finalmente no son consumidos?<br>La mayor parte de este desperdicio lo producimos nosotros, los consumidores, en nuestras casas.<br>Guardar las sobras de comidas para comerlas en otra ocasión, conservar bien los alimentos, o consumir primero los alimentos que llevan más tiempo en casa para evitar que se echen a perder son algunas de las estrategias para reducir el desperdicio de comida.<br>A continuación encontrarás un enlace por si quieres descubrir diferentes formas para reducir el desperdicio alimentario desde tu propia casa. ¿Cuántas de ellas vas a poner en práctica?<br>https://www.eufic.org/es/seguridad-alimentaria/articulo/consejos-para-reducir-el-desperdicio-de-alimentos/

		5.4 Information about social consequences 4.1 Instructions on how to perform a behavior	All foods have lives of people behind. When we hear about sustainable diets, many times the only thing that comes to our minds is the environmental impact of food. However, sustainability includes not only the environmental dimension of food, but also the social and economic dimensions. The current food system is also socially unsustainable. There is a high rate of people suffering from unfair wage and working conditions, including forced labour and child exploitation.<br>To support a food system that is fair and sustainable, it is important to learn about the origin of food, how it has been produced, and whether all the people involved, from production to marketing, have decent working conditions. You can find out about the sustainability policy of your usual brands, as well as the shops where you buy your food. Have you ever thought about the socio-economic dimension of food sustainability?	Todos los alimentos tienen vidas de personas detrás.<br>Al oír hablar de dieta sostenible, muchas veces lo único que nos viene a la cabeza es el impacto ambiental de los alimentos. No obstante, la sostenibilidad no solo incluye la dimensión ambiental de la alimentación, sino también la social y económica.<br>El sistema alimentario actual también es insostenible desde este punto de vista social. Existe un alta tasa de personas que sufren unas condiciones salariales y laborales injustas, incluso está muy presente el trabajo forzado y la explotación infantil.<br>Para apoyar un sistema alimentario que sea justo y sostenible, es importante con nos informemos sobre el origen de los alimentos, cómo estos se han producido, y si todas las personas involucradas, desde la producción hasta la comercialización, tienen unas condiciones laborales dignas.<br>Puedes informarte sobre la política de sostenibilidad de tus marcas habituales, así como de los comercios donde adquieres tus alimentos.<br>¿Habías pensado alguna vez en la dimensión socio-económica de la sostenibilidad alimentaria?

	Week 11	4.1 How to perform a behavior	As you may already know, it is said that you have to take at least 5 servings of fruits and/or vegetables a day. Making a list of different options to incorporate them into your day to day can help. For example, add fruit to salads (e.g., oranges, strawberries, apples...), on breakfast toast (e.g., sliced banana), or as a dessert or snack; in the case of vegetables, you can use them to make sauces, or take them as a side dish. Make a list of all the ideas you can think of. In fact, you can make a list for all those healthy and sustainable foods that you are not used to taking in your day to day and want to include in your diet, such as legumes, whole grains or nuts.	Como quizás ya sepas, se dice que hay que tomar al menos 5 raciones de frutas y/o verduras al día. Hacer una lista con diferentes opciones para incorporarlas en tu día a día te puede ayudar. Por ejemplo, añadir frutas a las ensaladas (p.ej., naranjas, fresas, manzanas...), en las tostadas del desayuno (p.ej., plátano en rodajitas), o como postre, merienda o almuerzo; en el caso de las verduras, las puedes usar para hacer salsas, o tomarlas como guarnición. Haz una lista con todas las ideas que se te ocurran. De hecho, puedes hacer una lista para todos aquellos alimentos saludables y sostenibles que no estés acostumbrado a tomar en tu día a día y quieras incluir en tu dieta, como legumbres, cereales integrales o frutos secos.
		1.4 Action planing	If you have a dietary goal, clearly specifying how you’re going to accomplish it can help you achieve it. Make a detailed plan of where, how and how often you will do that action. For example, order a salad as a substitute for fries every time you eat out, or have fruit for dessert at lunches and dinners Monday through Friday. Writing it down or telling a friend or family member can reinforce your commitment to achieving your goal, and don’t give up. So you know, set your goal clearly, and... go for it!	Si tienes un objetivo dietético, especificar claramente cómo lo vas a llevar a cabo puede ayudarte a alcanzarlo. Haz un plan detallado de dónde, cómo y con qué frecuencia harás esa acción. Por ejemplo, pedir una ensalada como sustituto de patatas fritas cada vez que salgas a comer, o tomar fruta de postre en las comidas y cenas de lunes a viernes. Ponerlo por escrito o contárselo a algún amigo o familiar puede reforzar tu empeño en alcanzar tu objetivo, y que no desistas hasta conseguirlo. Así que ya sabes, establece tu objetivo claramente, y… ¡a por él!

	Week 12	5.4 Information about environmenta l and social consequences	You have probably heard of eating seasonal fruits and vegetables to reduce the environmental impact of your diet. And it’s true. To be able to have a food available outside its usual season, you have to artificially create the specific climatic conditions that that product needs in a greenhouse (temperature, light...) and this, of course, requires energy; an energy that is not necessary when it is grown in a field in the proper season for that food.<br>But the benefits of seasonal fruits and vegetables don’t end there: it’s the moment when they’re tastier and cheaper!! In the next screen I will find a link to a table that shows when different fruits and vegetables are in season in Catalonia in case you do not have it very clear. Are you ready to save some money?https://agricultura.gencat.cat/web/.content/04-alimentacio/cadena-alimentaria/codi-bones-practiques-comercials/documents/fitxers-binaris/calendari-productes-temporada.pdf	Probablemente hayas oído hablar de comer frutas y verduras de temporada para reducir el impacto ambiental de tu alimentación. Y es cierto.<br>Para poder tener disponible un alimento fuera de su temporada habitual, hay que crear artificialmente las condiciones climáticas específicas que necesita ese producto en un invernadero (temperatura, luz…) y esto, por supuesto, requiere energía; una energía que no es necesaria cuando son cultivados en un campo en la temporada propia para ese alimento.<br>Pero los beneficios de las frutas y verduras de temporada no terminan ahí: ¡¡es el momento justo en el que son más sabrosas y más baratas!!<br>En la siguiente pantalla te dejo un link a una tabla que muestra cuándo diferentes frutas y verduras están de temporada en Cataluña por si no lo tienes aún muy claro.<br>¿Estás listo/a para ahorrarte unos dinerillos?<br>https://agricultura.gencat.cat/web/.content/04-alimentacio/cadena-alimentaria/codi-bones-practiques-comercials/documents/fitxers-binaris/calendari-productes-temporada.pdf

		5.4 Information about environmenta l and social consequences	Had you ever thought that with your food choices you could contribute to fixing employment in your environment?<br>Effectively, buying locally produced food you are favoring the local economy as well as the setting of jobs in your environment. Similarly, shopping in local markets and small shops in your neighborhood also help to these small traders to subsist in front of large companies.<br>Do you usually look at the origin of your food? Do you frequent local markets?	¿Te habías parado a pensar alguna vez que con tus decisiones alimentarias pudieras contribuir a la fijación de empleo en tu entorno?<br>Efectivamente, comprando alimentos de producción local estás favoreciendo la economía local, así como la fijación de empleo en tu entorno. De igual manera, hacer tus compras en mercados locales y comercios del barrio también contribuye a que estos/as pequeños/as comerciantes, muchas veces a su vez abastecidos/as por productores/as locales, puedan subsistir frente a grandes superficies.<br>¿Te sueles fijar en la procedencia de tus alimentos? ¿Frecuentas mercados locales?
Month 4	Week 13	7.1 Prompts/cues	Visual reminders can help you achieve your goal. For example, having the image of the earth as a mobile or computer desktop wallpaper may remind you of the need to follow a sustainable diet to fight the climate crisis; an image of a little lamb or piglet, your commitment to reducing your meat consumption; or an image of a fruit-filled fruit bowl, your goal is to increase its consumption.<br>Do you think this would be useful for you? Try it!	Los recordatorios visuales pueden ayudarte a conseguir tu objetivo. Tener la imagen de la tierra como fondo de pantalla del móvil o del escritorio del ordenador, por ejemplo, puede recordarte la necesidad de seguir una dieta sostenible para luchar contra la crisis climática; una imagen de un corderito o cerdito, tu compromiso con reducir tu consumo de carne; o una imagen de un frutero repleto de frutas, tu objetivo de incrementar su consumo.<br>¿Crees que esto sería útil para ti? ¡Pruébalo!

		5.1 & 5.4 Information about health and environmenta l consequences	You might think that the consumption of dairy is essential to take calcium, but foods such as broccoli, almonds, white beans, sesame, soy drink enriched in calcium or tofu are excellent sources of calcium, and with a much lower environmental impact than dairy. In fact, to follow a healthy and sustainable diet, the consumption of dairy (in case you want to consume them, since they are not essential in our diet) must be moderate, the equivalent of a glass of milk a day or two fingers of cheese.	Es posible que siempre hayas creído que los lácteos son la única fuente de calcio, pero alimentos como el brócoli, almendras, alubias blancas, sésamo, la bebida de soja enriquecida en calcio o tofu son excelentes fuentes de calcio, y con un impacto ambiental mucho menor que los lácteos.<br>De hecho, para seguir una alimentación saludable y sostenible, el consumo de lácteos (en caso de que se quieran consumir, ya que no son imprescindibles en nuestra alimentación) debe ser muy moderado, el equivalente a un vaso de leche al día o dos dedos de queso.
	Week 14	6.2 Social comparison	Some of the most common goals people set when starting to follow a healthy and sustainable diet are:<br>Eat legumes daily, at lunch or at dinner.<br>Eliminate, or at least reduce, sausages and cheese.<br>Incorporate fruits and/or vegetables in each of the 5 main meals.<br>Take advantage of all the leftovers in the following meals, storing the leftovers at home and asking to be put to take what you have left in the restaurants.<br>Consume less soda and unhealthy snacks.<br>Do they match yours or have you set other goals?	Algunas de las metas más habituales que la gente se propone al comenzar una dieta saludable y sostenible son:<br>Consumir legumbres de manera diaria, en la comida o en la cena.<br>Eliminar, o al menos reducir, los embutidos y el queso.<br>Incorporar frutas y/o verduras en cada una de las 5 comidas principales.<br>Aprovechar todas las sobras en las siguientes comidas, guardando la comida que le sobra en casa y pidiendo que le pongan para llevar lo que le sobra en los restaurantes.<br>Consumir menos refrescos y snacks poco saludables.<br>¿Coinciden con las tuyas o te has planteado otras metas?

		5.4 Information about environmenta l consequences	You have probably heard that to reduce the carbon footprint of our food it is essential to consume local products. Is this true?<br>Although common sense tells us that transport is what mainly determines the carbon footprint of a food, this is not as that. Transport contributes less than 10% of the greenhouse gas emissions (those responsible for climate change) of a food. The carbon footprint of a food is mainly determined (70-80%) by the type of product it is (i.e., whether it is a vegetable, legume, chicken or lamb).<br>The benefit of eating local food is socio-economic (the local economy is promoted and job fixing in the environment), but the environmental benefit is minimal.	Es probable que hayas oído que para reducir la huella de carbono de nuestra alimentación es fundamental consumir productos locales. ¿Es esto cierto?<br>Aunque el sentido común nos indique que el transporte es lo que principalmente determina la huella de carbono de un alimento, esto no es así. El transporte contribuye en menos de un 10% de las emisiones de gases de efecto invernadero (los responsables del cambio climático) de un alimento. La huella de carbono de un alimento viene determinada principalmente (70-80%) por qué producto es (es decir, si es una verdura, legumbre, carne de pollo o carne de cordero).<br>El beneficio de comer local es socio-económico (se promueve la economía local y la fijación de empleo en el entorno), pero el beneficio ambiental es mínimo. Comer un filete de ternera de producción local tendrá una huella de carbono muy superior al de alubias traídas desde la otra parte del mundo.<br>Por ello, si tu propósito es reducir el impacto ambiental de tu alimentación, fíjate en qué comes más que de dónde viene. Reducir el consumo de carne y lácteos es clave para reducir el impacto ambiental de tu alimentación. Eating a locally-produced beef steak will have a much higher carbon footprint than beans brought from the other side of the world. So if your purpose is to reduce the environmental impact of your diet, look at what you eat more than where it comes from. Reducing meat and dairy consumption is key to reducing the environmental impact of your diet.

	Week 15	3.1 Social support	Friends, family, or coworkers can help you reach your dietary goals. For example, tell them that you are trying to follow a healthier and more sustainable diet. Expressing it to others can be a way to increase your commitment to achieving your dietary goal.<br>Have you made your dietary goals public?	Amigos, familiares o compañeros de trabajo pueden ayudarte a que alcances tus propósitos dietéticos. Por ejemplo, coméntales que estás tratando de seguir una alimentación más saludable y sostenible. Expresarlo frente a otros puede ser una forma de incrementar tu compromiso para alcanzar tu objetivo dietético.<br>Y tú, ¿ya has hecho públicos tus objetivos dietéticos?
		6.1 Demostration of the behavior Recipe	Eating legumes or tofu instead of animal protein sources -which are not as healthy and have a greater impact on the environment- doesn't have to be difficult or boring. In salads, in the form of vegetable creams or pâtés, legume pasta, or simply a stew. The variety of options is endless! See how others prepare them can inspire you and you can get ideas so you can incorporate their consumption in your day to day in an appetizing way. Try searching for videos or recipes online, or simply asking your friends and family. Meanwhile, you can start with the recipe of chickpea meatballs that we leave you in the following link:: https://drive.google.com/file/d/16jsRfubvnFMXxYlSjZbRkMFxD9au7/view?usp=sharing	Consumir legumbres o tofu en lugar de fuentes proteicas de origen animal -que no son tan saludables y suponen un mayor impacto en el medioambiente- no tiene porqué resultar difícil o aburrido. En ensaladas, en forma de cremas o patés vegetales, pasta de legumbres, o simplemente el potaje de toda la vida. ¡La variedad de opciones es infinita! Ver cómo otros las preparan te puede inspirar y puedes coger ideas para que puedas incorporar su consumo en tu día a día de manera apetitosa. Prueba buscando vídeos o recetas en internet, o simplemente preguntando a tus amigos y familiares.<br>Mientras tanto, puedes comenzar a abrir boca con la receta de albóndigas de garbanzo que te dejamos en el siguiente link:<br>https://drive.google.com/file/d/16jsRfubvcgnFMXxYlSjZbRBkMFxD9au7/view?usp=sharing

	Week 16	1.1 Behavioral goal setting	If you think your consumption of any type of food is excessive or insufficient, and you want to reduce or increase it, having a clear goal can help you do so. Set a dietary goal as concrete as possible, specifying the amount (e.g. portions or frequency of consumption) as well as the period during which you will do it (e.g. this week, this month). This goal should be realistic depending on how you eat normally. For example, if you eat red or processed meat 4 days a week, consider eating it only two days a week during this month. Once the goal is reached, go one step further; for example, eat red meat or processed only one day a week for the next month.<br>What would you like to change your diet from today?	Si crees que tu consumo de algún tipo de alimento es excesivo o insuficiente, y quieres reducirlo o aumentarlo, tener una meta clara puede ayudarte a hacerlo.<br>Establece un objetivo dietético lo más concreto posible, especificando la cantidad (p.ej. porciones o frecuencia de consumo) así como el período durante el que lo llevarás a cabo (p.ej. esta semana, este mes). Este objetivo debe ser realista en función de cómo comes normalmente.<br>Por ejemplo, si comes 4 días a la semana carne roja o procesada, plantéate comerla solo dos días a la semana durante este mes.<br>Una vez alcanzado el objetivo, ve un paso más allá; por ejemplo, comer carne roja o procesada solo un día a la semana durante el próximo mes.<br>Y a ti, ¿qué te gustaría cambiar de tu alimentación desde hoy mismo.
		4.1 Instructions on how to perform a behavior	Did you know that distinguishing between expiration date and best by date can be key to reducing food waste? The expiration date indicates the date from which a food should NOT be eaten as it supposes a toxicological risk; it usually appears in fresh and highly perishable foods such as meat and fish. On the other hand, the best by date, which usually appears in yogurts, frozen products, cans, and packaged products in general, indicates the date until which the characteristics of flavor, smell, texture... is guaranteed. However, once this date has passed and if the food has been preserved in the right conditions, there is no problem in consuming it once this date has expired. Look at it, smell it and taste it. If nothing tells you otherwise, you can consume it without any risk to your health. That is, it can be consumed perfectly and therefore, it is not necessary to throw it away.	¿Sabías que distinguir entre fecha de caducidad y fecha de consumo preferente puede ser clave para reducir el desperdicio alimentario?<br>La fecha de caducidad indica la fecha a partir de la cual un alimento NO se debe comer ya que supone un riesgo toxicológico; suele aparecer en alimentos frescos y altamente perecederos como carne y pescado. En cambio, la fecha de consumo preferente, que suele aparecer en yogures, productos congelados, conservas, y productos envasados en general, indica la fecha hasta la que las características de sabor, olor, textura... se mantienen intactas. Sin embargo, una vez superada esta fecha y si el alimento se ha conservado en las condiciones adecuadas, no hay ningún problema en consumirlo una vez vencida esta fecha. Míralo, huélelo y pruébalo. Si no hay nada que te

				indique lo contrario, puedes consumirlo sin que suponga ningún riesgo para tu salud. Es decir, una vez superada la fecha de consumo preferente puede ser consumido perfectamente y por tanto, no es necesario tirarlo a la basura.
Month 5	Week 17	4.1 Instructions on how to perform a behavior	It is quite obvious that bakery products or sweets contain a lot of sugar, and that it is advisable to avoid them. However, foods that we consume in our day to day, such as cookies, breakfast cereals, flavored yogurts or many sauces, can contain a lot of sugar and we are not aware of it. In order to detect these high-sugar foods, look at the nutrition label. If they have more than 10 grams of sugar per 100 grams of product, it indicates that it is a food high in sugars, and that it should be avoided. In fact, the ideal is to consume foods whose sugar content is less than 5g per 100g of product. You can consult the website sinazucar.org and you will be surprised by all the hidden sugar that certain foods have	Es bastante obvio que productos de bollería o las chucherías contienen gran cantidad de azúcar, y que conviene evitarlos. Sin embargo, alimentos que consumimos en nuestro día a día, como las galletas, los cereales de desayuno, los yogures de sabores o muchas salsas, pueden contener gran cantidad de azúcar y no somos conscientes de ello.<br>Para poder detectar estos alimentos altos en azúcar, mira la etiqueta nutricional. Si tiene más de 10 gramos de azúcar por cada 100 gramos de producto, indica que es un alimento alto en azúcares, y que conviene evitarlo. De hecho, lo ideal es consumir alimentos cuyo contenido en azúcar sea menor de 5g por cada 100g de producto.<br>Puedes consultar la web sinazucar.org y te sorprenderá toda la azúcar oculta que tienen los alimentos
		Restructuring the physical environment	Your environment can help or hinder your transition to a healthier and more sustainable diet. For example, having a fruit bowl with different fruits always in sight can help you to consume more fruit; if you try to reduce sweets consumption, limit their availability at home as much as possible, or at least keep them out of sight; or always carry nuts or fruit to avoid buying unhealthy products if you get hungry outside your home. Think, what small changes in your environment do you think can help you?	Tu entorno puede ayudar o dificultar tu transición hacia una dieta más saludable y sostenible. Por ejemplo, tener un frutero con diferentes frutas siempre a la vista puede ayudarte a consumir más frutas; si intentas reducir los dulces, limita su disponibilidad en casa al máximo, o al menos mantenlos fuera de la vista; o llevar siempre encima frutos secos o fruta para evitar comprar productos malsanos si te entra hambre fuera de casa. Piensa: ¿qué pequeños cambios de tu entorno crees que pueden ayudarte?

	Week 18	Recipe	If you are one of those who do not know what to have for breakfast beyond breakfast cereals, cookies and toast, I leave here a healthy recipe, with low environmental impact, cheap, fast to make and delicious. In a delicious way you can incorporate whole grains, fruit and nuts in your day to day. I would try it ;)<br>https://drive.google.com/file/d/1uTdmBL0smla83-T_cnl2vj7ALzUWX1ZA/view?usp=sharing	Si eres de los que no sabes qué desayunar más allá de los cereales de desayuno, las galletas y las tostadas, a continuación encontrarás una receta saludable, con bajo impacto ambiental, barata, rápida de realizar y muy rica. De una manera deliciosa podrás incorporar cereales integrales, fruta y frutos secos en tu día a día. Yo que tú la probaría ;)<br>https://drive.google.com/file/d/1uTdmBL0smla83-T_cnl2vj7ALzUWX1ZA/view?usp=sharing
		5.1 Information about health consequences<br>4.1 Instructions on how to perform a behavior	A lot of the calories we take in our day to day are not intake through food, but by drinks, such as soft drinks, juices or alcoholic beverages. If you are going to consume these types of drinks, save them for special occasions, and not as a daily routine. Some more recommended alternatives would be, for example: sparkling water instead of soft drinks, sugarless infusion instead of juices, non-alcoholic beer instead of beer with alcohol. However, the best option will always be water. If you want, you can add a slice of lemon and/or a mint leaf to the water to give it some flavor. And if is it tap water even better: it is as healthy as bottled water, while cheaper and with less environmental impact.	Una gran cantidad de las calorías que tomamos en nuestro día a día no las ingerimos a través de los alimentos, sino por las bebidas, como refrescos, zumos o bebidas alcohólicas. Si vas a consumir este tipo de bebidas, resérvalas para ocasiones especiales, y no como una rutina diaria.<br>Algunas alternativas más recomendables serían, por ejemplo:<br>agua con gas en vez de refrescos<br>infusión sin azúcar en vez de zumo<br>cerveza sin alcohol en vez de con alcohol<br>No obstante, la mejor opción siempre será el agua, a la que si quieres puedes añadir una rodajita de limón y/o una hoja de menta para darle algo de sabor. Y si es del grifo mejor que mejor, que es igual de saludable, más barata y con un menor impacto ambiental.

	Week 19	5.1 Information about health consequences	There is a false belief that fats are bad for health. The problem is not fats; the problem is the TYPE of fat. Numerous studies talk about the cardiovascular benefits of virgin olive oil. Therefore, using virgin olive oil instead of other types of fats, such as sunflower or corn oil, margarine or butter, will make your diet a healthier pattern.	Existe una falsa creencia de que las grasas son malas para la salud. El problema no son las grasas; el problema es el TIPO de grasa. Por ejemplo, numerosos estudios hablan de los beneficios cardiovasculares del aceite de oliva virgen. Usar aceite de oliva virgen en lugar de otros tipos de grasas, como aceite de girasol o de maíz, margarina o mantequilla, hará de tu dieta un patrón más saludable.
		1.2 Problem solving	Many times, lack of time, strong eating habits, or social relationships can make it difficult for you to achieve the dietary goals you have set. To avoid this, it is important to identify what these barriers are and think about ways to overcome them. For example, "IF you do not consume legumes for lack of time to cook them in your day to day, THEN cook a big quantity and keep frozen individual servings". Solving problems this way (IF..., THEN...) can help you achieve your goals.<br>Do you dare to put this tip into practice?	Muchas veces, la falta de tiempo, los fuertes hábitos alimenticios, o las relaciones sociales pueden dificultar que alcances los objetivos dietéticos que te has propuesto. Para evitarlo, es importante que identifiques cuáles son estas barreras y pienses en maneras de superarlas. Por ejemplo, "SI no consumes legumbres por falta de tiempo para cocinarlas en tu día a día, ENTONCES cocina un día una gran cantidad y guarda raciones individuales congeladas". Resolver problemas de esta manera (SI..., ENTONCES...) puede ayudarte a alcanzar tus objetivos.<br>¿Te animas a poner este consejo en práctica?
	Week 20	4.1 How to perform a behavior	Do you know the 3 Rs rule of packaging?<br>1st REDUCE the use of packaging: avoid those foods that come with unnecessary packaging, such as packaged fresh fruits or vegetables, and prioritize bulk purchase<br>2nd REUSE: once purchased, you can give a second life to your packaging. Search online and you will find hundreds of ideas for reusing them.<br>3rd RECYCLE: separate waste so that it can be recycled.<br>But beside recycled has received all the attention, we must remember that the rules must be applied in this order, and that the main goal is to avoid the purchase of food that carry unnecesary packaging	¿Conoces la regla de las 3 Rs de los envases?<br>1ª REDUCIR el uso de envases: evita aquellos alimentos que vengan con envases innecesarios, como frutas o verduras frescas envasadas, y da prioridad a la compra a granel<br>2ª REUTILIZAR: una vez adquiridos, puedes dar una segunda vida a tus envases. Busca en internet y encontrarás cientos de ideas para reutilizarlos.<br>3ª RECICLAR: separa los residuos para que puedan ser reciclados.<br>Pero por mucho énfasis que se haya hecho en el reciclaje, las reglas se deben aplicar en este orden, siendo lo principal

				el evitar la adquisición de alimentos que lleven un envase innecesario.
		7.1 Prompts/cues	A few weeks ago we told you that having visual reminders (for example, the image of a planet in the mobile wallpaper) can help you remember your goal and stay on it. Remember to change it from time to time, as seeing it constantly can lead you to ignore it. Try changing it once a month (or as often as you think it may be more useful for you) or adding new ones (for example, putting a picture in the fridge of a healthy and sustainable meal that you have recently prepared and enjoyed).<br>What's the next reminder you will put in?	Hace unas semanas ya te dijimos que poner recordatorios visuales (por ejemplo, la imagen de un planeta en el fondo de pantalla del móvil) puede ayudarte a recordar tu objetivo y mantenerte en ello. Recuerda cambiarlo de vez en cuando, ya que verlo de manera constante puede hacer que llegues a ignorarlo. Prueba de cambiarlo una vez al mes (o con la frecuencia que tú consideres que puede ser más útil para ti) o añadir otros nuevos (por ejemplo, poner una foto en la nevera de una comida saludable y sostenible que hayas preparado recientemente y la hayas disfrutado).<br>¿Cuál va a ser el próximo recordatorio que pongas?
Month 6	Week 21	2.3 Self-monitoring	Some time ago we asked you to write down your food consumption over the course of a week. We encourage you to do it again, so you can assess how your diet has changed during these months. How is your consumption of red and processed meats? and dairy? and fruits and vegetables? and highly processed foods high in sugars? Doing this self-monitoring from time to time can help you become aware of where you are and be able to detect aspects that you would like to improve.	Ya hace un tiempo te pedimos que fueras anotando tu consumo de alimentos a lo largo de una semana. Te animamos a que lo vuelvas a hacer, y así podrás valorar cómo ha cambiado tu alimentación durante estos meses. ¿Cómo es tu consumo de carnes rojas y procesadas? ¿y de lácteos? ¿y de frutas y verduras? ¿y de alimentos muy procesados altos en azúcares?<br>Hacer este auto-monitoreo de tanto en tanto puede ayudarte a tomar conciencia de en qué punto estás y poder detectar aspectos que te gustaría mejorar.

		4.2 Obtain information about antecedents	It is possible that during these months you have set yourself some dietary goals. Have you reached them? If so, what helped you? And when you didn't reach them, what made it difficult? Keeping facilitators and barriers in mind can help you reach future goals.	Es posible que a lo largo de estos meses te hayas marcado algún que otro objetivo dietético. ¿Los has alcanzado? Si ha sido así, ¿qué te ha ayudado a lograrlo? Y cuando no los alcanzaste, ¿qué es lo que lo dificultó? Tener presente tanto los facilitadores como las barreras puede ayudarte a alcazar futuros objetivos.
	Week 22	15.3 Focus on past success	If you ever find yourself returning to your old eating habits and want to continue to feed yourself in a healthy and sustainable way, thinking about past successes can give you the confidence to do the same again. Think of moments in the past when you set out to eat healthier and more sustainably. Think about how you did it and how things were back then. For example, you set out to eat legumes every day at one of the main meals (lunch or dinner). You made it, but in the last two weeks you've only eaten them twice for lack of time. You managed to reach your goal by always having at home canned chickpeas, lentils and white beans, individual portions of frozen cooked legumes, as well as lentil macaroni. So if you forgot to soak dry legumes the night before or didn't have time to cook, you always had quick options available. Returning to this practice could help you reach your goal of consuming legumes daily.<br>Have you noticed that you have returned to some old eating habit that you would like to change? If so, think about it and find the options to get it that best suit you. If not, if every day you eat healthier and more sustainable, congratulations! Keep it up!	Si en algún momento ves que estás volviendo a tus viejos hábitos alimentarios y quieres continuar alimentándote de manera saludable y sostenible, pensar en éxitos pasados puede darte confianza para hacer lo mismo de nuevo. Piensa en momentos del pasado donde te propusiste y lograste comer de manera más saludable y sostenible. Piensa en cómo lo hiciste y cómo eran las cosas en esos tiempos.<br>Por ejemplo, te propusiste comer legumbres todos los días en una de las comidas principales (comida o cena). Lo conseguiste, pero en las últimas dos semanas solo las has comido dos veces por falta de tiempo. Conseguiste alcanzar tu objetivo teniendo siempre en casa conservas de garbanzos, lentejas y alubias blancas, raciones individuales de legumbres ya cocinadas congeladas, así como macarrones de lentejas. De manera que si la noche de antes te olvidabas de poner las legumbres secas a remojo o no tenías tiempo para cocinar, siempre tenías opciones rápidas disponibles. Volver a esta práctica te podría ayudar a volver a alcanzar tu objetivo de consumir legumbres diariamente

				Y tú, ¿has notado que has vuelto a algún viejo hábito alimentario que te gustaría cambiar? Si es así, piensa en ello y encuentra las opciones para conseguirlo que más se adapten a ti. Si no, si cada día comes más saludable y sostenible, ¡enhorabuena!, sigue así
		5.4 Information about environmental and social consequences	And what about other drinks like coffee and tea? Often these products are grown through agricultural practices that have a huge environmental impact, and the working conditions of workers are very poor, even resorting to forced labor and child exploitation. Therefore, if you consume these products, opt for those with labels that ensure that practices used were respectful to both the environment and producers. Some examples of these labels in the case of coffee, cocoa and tea are rainforest allianze, UTZ or fairtrade.<br>Have you ever noticed these labels? From now on you will, for sure!	¿Y qué pasa con otras bebidas como el café y el té? Muchas veces estos productos se cultivan mediante prácticas agrícolas que tienen un enorme impacto ambiental, y siendo las condiciones laborales de los trabajadores pésimas, recurriendo incluso a trabajos forzados y explotación infantil. Por ello, si consumes estos productos, opta por aquellos con sellos que garanticen que se han obtenido mediente prácticas respetuosas tanto con el medioambiete como con los productores. Algunos ejemplos de estos sellos en el caso del café, cacao y té son rainforest allianze, UTZ o fairtrade.<br>¿Te habías fijado alguna vez en estas etiquetas? Seguro que a partir de ahora sí que lo harás
	Week 23	4.1 How to perform a behavior Recipe	Would you like to try a simple and delicious recipe with which to incorporate whole grains and nuts in your diet? Two of the food groups that should not be missed in your day to day, along with fruits, vegetables and legumes, to follow a healthy and sustainable diet. Discover it in the following link. Bon Appetit!<br>https://drive.google.com/file/d/1uTdmBL0smla83-T_cnl2vj7ALzUWX1ZA/view?usp=sharing	¿Te apetece probar una receta sencilla y deliciosa con la que incorporar cereales integrales y frutos secos en tu alimentación? Dos de los grupos de alimentos que no debieran faltar en tu día a día, junto con las frutas, verduras y legumbres, para seguir una alimentación saludable y sostenible.<br>Descúbrela en el siguiente enlace.<br>¡Buen provecho!<br>https://drive.google.com/file/d/1uTdmBL0smla83-T_cnl2vj7ALzUWX1ZA/view?usp=sharing

		5.6 Information about emotional consequences	If you look back and think about what your diet was like when the study started and how it is now, do you see any changes? If yes, how do you feel about yourself after these changes? Do you think it has been positive? If so, do you want to maintain your new dietary pattern over time? Would you change anything else about your diet? If so, what could you do to achieve this change?<br>Making this visualization will make you aware of all the benefits that your food change has had (improvements in your health, reduction of environmental impact, obtaining food from fair and ethical sources, decrease in animal exploitation...) and will strengthen you to stay in them.	Si echas la vista atrás y piensas en cómo era tu alimentación cuando comenzó el estudio y cómo es ahora, ¿aprecias algún cambio?<br>Si es que sí, ¿cómo te sientes contigo mismo/a tras estos cambios? ¿crees que ha sido positivo?<br>Si es así, ¿quieres mantener tu nueva forma de alimentarte en el tiempo?<br>¿Cambiarías algo más de tu alimentación?<br>Si es así, ¿qué podrías hacer para lograr este cambio?<br>Hacer esta visualización te hará tomar conciencia de todos los beneficios que ha tenido tu cambio alimentario (mejoras en tu salud, reducción del impacto ambiental, obtención de alimentos de fuentes más justa y éticas, disminución de la explotación animal…) y te reforzará para mantenerte en ellos.
	Week 24	12.1 Restructuring the physical environment	You probably have already acquired the habit of eating healthy and sustainable foods in your day to day, some of them you were not used to consume them previously, without the necessity of thinking too much about it. However, keep in mind that having an environment that favors this way of eating, such as always carrying fruit away from home or avoiding having unhealthy food at home, can always be an extra help to keep you in it.	Probablemente ya hayas adquirido el hábito de comer en tu día a día alimentos saludables y sostenibles a los que antes no estabas acostumbrado/a, sin que te suponga ya un esfuerzo y no necesites recordártelo constantemente. No obstante, ten presente que tener un entorno que favorezca esta forma de alimentarse, como llevar siempre fruta fuera de casa o evitar tener comida malsana al alcance, siempre puede ser una ayuda extra para mantenerte en ello.

		Farewell	Congratulations! You have already spent half a year in the study "S2 eating: for a healthy and sustainable diet". We hope that participating in the study is being useful to understand the relevance of healthy and sustainable eating, and how to implement it. From now on you will no longer receive text messages, nor having the feedback meetings, but we hope to continue to count on your participation in the rest of the study. THANK YOU AGAIN FOR YOUR PARTICIPATION	¡Enhorabuena! Ya llevas medio año en el estudio "Alimentación S2: por una dieta saludable y sostenible". Esperamos que la participación en el estudio te esté siendo útil para comprender la relevancia de llevar una alimentación saludable y sostenible, y cómo llevarla a la práctica. A partir de ahora ya no recibirás más mensajes de texto, ni tendremos las sesiones de seguimiento, pero esperamos seguir contando con tu participación en lo que resta de estudio. GRACIAS DE NUEVO POR FORMAR PARTE DEL ESTUDIO
