# Supplementary material for: A Smartphone Intervention to Promote a Sustainable Healthy Diet: Protocol for a Pilot Study
Source: JMIR Res Protoc. 2023 Mar 2;12:e41443. doi: 10.2196/41443 (PMC10020902; doi:10.2196/41443)
Supplement: Multimedia Appendix 4 [file resprot_v12i1e41443_app4.pdf]

Supplemental material 4: Baseline questionnaire (English and Spanish versions)

Category	Name	Item	Answer options
Socio-demographic	Gender	Gender	1. Female 2. Male 3. Other 4.Prefer not to say
	Age	Age	Numeric value (years old)
	Education level	Higher level of education achieved	1. No studies 2. Primary (EGB, primaria) 3. Secundary (ESO, COU, bachillerato, FP I, FP II, ciclo formativo de grado medio o superior) 4. University (Diplomatura, licenciatura, grado, ingeniería técnica o superior) 5. Master or postgraduate studies 6. Doctorate
	Incomes	Monthly incomes (after taxes) of the family unit	<500, 500–999, 1000–1499, 1500–1999, 2000–2999, 3000–3999,≥4000 euros after tax
	Political orientation	What political position do you identify with?	1. Extreme left 2. Left 3. Center left 4. Center 5. Center right 6. Right 7. Extreme right
	Postal code	Postal code of your home	Numeric value
	cohabitation	Apart from you, how many people do you live with?	Numeric value
	cooking for someone	If you usually cook, do you cook only for yourself, or also for other people?	1. Just for me 2. For other people too 3. I do not usually cook
Anthropometric	Height	How tall are you (in meters)?	Numeric value
	Weight	Which is your weight (in kg)?	Numeric value
Health	Self-perceived health	In general, how would you say your physical health is?	VAS 1-10<br>1-Extremelly bad 10-Excellent
	Self-perceived health	In general, how would you say your mental health is?	VAS 1-10<br>1-Extremelly bad 10-Excellent
	Supplements	Do you take supplements?	1. Yes 2. No
	Tobacco	Do you smoke?	1. I have never smoked; 2. I stopped smoking from more than 6-month; 3. Occasionally; 4. Everyday

Motivation	Environmental relevance	For you, protecting the environment and conserving nature is something…	VAS 1-10<br>1. Not important at all 10. Very important
	Auto-eficacy	I believe I can contribute to the fight against climate change	VAS 1-10<br>1. In total disagreement 10. In total agreement
	Climate change worry	How worried about climate change are you?	VAS 1-10<br>1. No worried at all 10. Extremely worried
	Personal norms	To what extent do you feel that trying to mitigate climate change is also your personal responsibility?	VAS 1-10<br>1. In total disagreement 10. In total agreement
	Risk perception	How likely is it that you are already experiencing, or that you will do so throughout your life, negative consequences for your health and well-being as a result of climate change?	VAS 1-10<br>1. Totally unlikely 10. Totally likely

VAS: visual analog scale

**Categoría**	**Nombre**	**Item**	**Opciones de respuesta**
Socio-demográfica	Género	Género	1. Femenino 2. Masculino 3.Otro 4.Prefiero no decirlo
	Edad	Edad	Valor numérico (años)
	Estudios	Nivel de estudio más alto alcanzado	1. Sin estudios 2. Primarios (EGB, primaria) 3. Secundarios (ESO, COU, bachillerato, FP I, FP II, ciclo formativo de grado medio o superior) 4. Universidad (Diplomatura, licenciatura, grado, ingeniería técnica o superior) 5. Máster o posgrado 6. Doctorado
	Ingresos	Ingresos mensuales (en neto) de la unidad familiar	<500, 500–999, 1000–1499, 1500–1999, 2000–2999, 3000–3999,≥4000 euros netos al mes
	Orientación política	¿Con qué posición política te identificas?	1. Extrema izquierda 2. Izquierda 3. Centro izquierda 4. Centro 5. Centro derecha 6. Derecha 7. Extrema derecha
	Código postal	Código postal de su residencia habitual	Valor numérico
	Convivencia	¿Cuántas personas viven en tu casa, además de ti?	Valor numérico
	Cocinar para otros	Si cocinas en tu día a día ¿lo haces solo para ti, o también para otras personas?	1. Solo para mí 2. Cocino también para otros 3. No suelo cocinar
Antropométrica	Altura	¿Cuál es tu altura (en metros)?	Valor numérico
	Peso	¿Cuánto pesas (en kg)?	Valor numérico
Salud	Salud auto-percibida	En general, ¿cómo dirías que es tu salud física?	EVA 1-10<br>1-Extremadamente mala 10-Excelente
	Salud auto-percibida	En general, ¿cómo dirías que es tu salud mental?	EVA 1-10<br>1-Extremadamente mala 10-Excelente
	Suplementos	¿Tomas algún suplemento alimenticio de manera continua?	1- Sí 2-No
	Tabaco	¿Eres fumador/a?	1. Nunca he fumado; 2. Dejé de fumar hace más de 6 meses; 3. Ocasionalmente; 4. Diariamente
Motivación	Valor medioambiental	Para ti, proteger el medioambiente y conservar la naturaleza es algo…	EVA 1-10<br>1. Nada importante 10. Muy importante

	Auto-eficacia	Creo que puedo contribuir en la lucha contra el cambio climático	EVA 1-10<br>1. Totalmente en desacuerdo 10. Totalmente de acuerdo
	Angustia cambio climático	¿Cómo de preocupado/a estás por el cambio climático?	EVA 1-10<br>1. Nada preocupado 10. Extremadamente preocupado
	Normas personales	¿En qué medida sientes que intentar mitigar el cambio climático es también una responsabilidad personal tuya?	EVA 1-10<br>1. Totalmente en desacuerdo 10. Totalmente de acuerdo
	Percepción de riesgo	A tu parecer, ¿cómo de probable es que ya estés experimentando, o que lo hagas a lo largo de tu vida, consecuencias negativas para tu salud y bienestar como resultado del cambio climático?	EVA 1-10<br>1. Totalmente improbable 10. Muy probable

EVA: escala visual analógica
