# Supplementary material for: A Smartphone Intervention to Promote a Sustainable Healthy Diet: Protocol for a Pilot Study
Source: JMIR Res Protoc. 2023 Mar 2;12:e41443. doi: 10.2196/41443 (PMC10020902; doi:10.2196/41443)
Supplement: Multimedia Appendix 5 [file resprot_v12i1e41443_app5.pdf]

Supplemental material 5: Supportive material showing specific food items gathered in the assessed food groups, and examples of one and half servings (English and Spanish versions).

Food groups		Specific foods included	Example of 1 serving	Example of ½ serving
Red and/or processed meat	Red meat	Beef, pork and lamb, offals (liver, kidney, etc.)	1 steak of the size of your palm<br>3 pieces of loin<br>3 meatballs	1 piece of loin (for example, added to the pasta sauce)
	Processed meat	Any type of cold-cuts, cured ham, cooked chicken or turkey ham/breast, any type of sausage, burgers, etc.	2 small sausages<br>3 slices of bacon<br>3 slices of cured ham<br>6 slices of chorizo, salami	1-2 small slices of cured ham<br>3 slices of fuet
White meat, fish and/or eggs	White meat	Chicken, turkey, rabbit	1 breast the size of your hand<br>1 medium size chicken leg<br>1/4 rabbit	1 big breast is equivalent to 1.5 servings
	Fish	Fresh, frozen, canned, smoked fish and seafood, octopus, cuttlefish, squid...	1 fish filet<br>1 medium size plate of prawns<br>1 medium size can of canned fish	1 individual can of tuna
	Eggs	In any format: boiled, fried, omelette...	1 egg	

Dairy products	Dairy products	Milk, yogurt, cheese, etc.<br>NO included: sugary dairy desserts like flan, custard, ice cream…	1 cup of milk<br>1 individual yogurt<br>2-3 slices of cheese; 2 spoons of spreadable cheese; 4 spoons of grated cheese	Milk added to coffee<br>1 slice of cheese
Legumes and soy products	Legumes and soy products	Any type (chickpeas, lentils, beans, etc.) and soy derivatives such as tofu or textured soy.	1 deep plateo f cooked legumes<br>1 block of the size of you palm of tofu<br>5 spoons of dried texturized soy protein	3-4 spoons of cooked legumes (for example, added into a salad)<br>1 really big deep place is equivalent to 1.5 servings
Fruits and/or vegetables	Fruits	Any type, fresh, frozen, bottled, canned…<br>NO included: fruit juices, jams, comercial yogurts with fruits	1 medium size unit (orange, apple, banana...)<br>2 small units (mandarines, kiwis...)<br>1 dessert plate (strawberries, berries, grapes, figs, higos...)<br>1 slice (melon, watermelon, pineapple...)	1 mandarin<br>3 strawberries
	Vegetables	Salads, raw or cooked vegetables, fresh, canned or frozen vegetables, peas, mushrooms, tomato sauce, gazpacho<br>NO included: potatoes, other tubers	1 plate of salad, cooked vegetables or puree.<br>1 big tomato<br>2 carrots<br>1 glass of gazpacho	1 carrot<br>2 spoons of tomato sauce

Nuts and seeds	Nuts and seeds	Any kind as long as they are roasted or natural (nuts, almonds, peanuts, cashews, pistachios, pumpkin seeds, sunflower, chia, etc.) and nut/seed butters without added sugar (peanut butter, tahini, etc.)<br>NO included: those nuts/seeds fried, with chocolate, sugar or any other coat, sugary chocolate spreads, with added sugars, dried fruits.	1 handful of shelled nuts/seeds<br>1 spoon of nut butter with no added sugar<br>1 spoon of chia seeds	2 walnuts<br>1 teaspoon of seeds
Grains[1]	Grains	Bread, pasta, rice, oats<br>NO included: commercial breakfast cereals, as they are considered as ultraprocessed foods	2 pieces of bread of the size of 4 fingers or 1 bun<br>1 deep dish of rice/pasta<br>4 spoons of oat flakes	2 rusks<br>1 really big deep place is equivalent to 1.5 servings
Ultraprocessed foods*	*Highly processed foods, rich in sugars, salt and/or fats.	Biscuits, pastry, (both homemade and industrial)<br>Snacks (crisps, candies, etc)<br>Sugary dairy desserts (custard, flan, etc)<br>Pre-cooked food (pizza, lasagna, etc)<br>Etc.	6 biscuits, 2 chocolate cookies<br>1 muffin, croisant<br>1 medium size piece of cake<br>1 small bag of crisps<br>1 individual protion of pizza, lasagna	2-3 biscuits<br>1 croqueta
Fats and oils[2]	Fats and oils	Virgin/extra virgin olive oil<br>Olive, sunflower, corn (etc.) oil<br>Butter<br>Margarine	1 spoon	

<table>
<tr><td>Alcoholic drinks</td><td>Alcoholic drinks</td><td>Beer, wine, spirits, etc.</td><td>1 bottle of beer
1 glass of wine
1 shot</td><td>1 beer with lemon equals to 1/2 serving of beer and 1/2 serving of soft drink</td></tr>
<tr><td>Soft drinks and other beberages</td><td>Soft drinks and other beverages</td><td>Soft drinks (with and without added sugars), juices (home-made and commercial), energy drinks, sodas, etc.</td><td>1 can of soft drink
1 glass of juice</td><td></td></tr>
<tr><td></td><td></td><td></td><td></td><td></td></tr>
<tr><td colspan="5">[1]How to measure the proportion of whole grains in your diet?</td></tr>
<tr><td colspan="5">You need to estimate the number of servings of whole grains (whole grain bread, brown pasta, brown rice, oats, etc.) you have consumed with respect to the total of cereals you have taken, and report the result as a percentage.
For example, if you have taken 1 serving of white rice at lunch, 1 serving of white bread during your afternoon snack, and 1 serving of whole grain bread at dinner, the proportion of whole grains will be 1 (serving whole grains)/3 (servings of grains total) = 0.33 -> 33% -> 33 de 100</td></tr>
<tr><td></td><td></td><td></td><td></td><td></td></tr>
<tr><td colspan="5">[2]How to measure the proportion of virgin/extre virgin olive oil in your diet?</td></tr>
<tr><td colspan="5">You need to estimate the number of servings of virgin/extra virgin olive oil you have consumed with respect to the total of servings of oils and fats (olive oil, sunflower oil, corn, oil, butter, margarine) you have taken during the day, and report the result as a percentage.
For example, if you have taken 1 serving of butter at breakfast, 1 serving of virgin olive oil at lunch, and 1 serving of virgin olive oil at dinner, the proportion of virgin olive oil will be 2 (servings of virgin olive oil)/3 (servings in total: 1 butter+2 virgin olive oil) = 0.66 -> 66% -> 66 de 100</td></tr>
</table>

**Grupo de alimentos**		**Qué incluye**	**Ejemplos de 1 ración**	**Ejemplos de 1/2 ración**
Carne roja y/o procesada	Carne roja	Carne de ternera, cerdo y cordero, vísceras (hígado, riñón, etc.)	1 filete del tamaño de la palma de tu mano<br>3 ruedas de lomo<br>3 albóndigas	1 rueda de lomo (por ejemplo, añadida a la salsa para pasta)
	Carne procesada	Embutidos de cualquier tipo, jamón serrano, jamón/pechuga cocido/a de pavo o pollo, salchichas de cualquier tipo, hamburguesas, etc.	2 salchichas pequeñas<br>3 lonchas de bacon<br>3 lonchas de jamón serrano<br>6 lonchas de chorizo, salami	1-2 lonchas pequeña de jamón serrano<br>3 rodajas de fuet
Carne blanca, pescado y/o huevos	Carne blanca	Carne de pollo, pavo, conejo	1 pechuga del tamaño de tu mano<br>1 muslo de pollo mediano<br>1/4 de conejo	1 pechuga grande contaría como 1 ración y 1/2
	Pescado	Pescado y marisco fresco, congelado, en lata, ahumado, pulpo, sepia, calamar…	1 filete de pescado<br>1 plato mediano de gambas<br>1 lata mediana de pescado en conserva	1 lata individual de atún
	Huevos	Cocinados de cualquier manera: cocido, frito, pasado por agua, en tortilla…	1 huevo	
Lácteos	Lácteos	Leche, yogur, queso, etc.<br>NO incluye postres lácteos azucarados como flan, natilla, helado…	1 taza de leche<br>1 yogur individual<br>2-3 lonchas queso; 2-3 tranchetes; 2 cucharadas de queso de untar; 4 cucharadas queso en polvo	Leche añadida al café<br>1 loncha de queso

Legumbres y derivados de soja	Legumbres	De cualquier tipo (garbanzos, lentejas, alubias, etc) y derivados de la soja como tofu o soja texturizada.	1 plato hondo normal de legumbres cocinadas<br>1 corte del tamaño de la palma de tu mano de tofu<br>5 cucharadas soperas en seco de proteína texturizada	3-4 cucharadas de legumbres cocidas (por ejemplo, añadidas a la ensalada)<br>1 plato muy grande representaría 1 ración y 1/2
Frutas y/u hortalizas	Frutas	De cualquier tipo, tanto frescas como congeladas, en conserva.<br>NO incluye zumo de frutas, mermeladas, yogur con frutas	1 pieza de tamaño medio (naranja, manzana, platano...)<br>2 pequeñas (mandarinas, kiwis...)<br>1 plato de postre (fresas, frutos rojos, uvas, higos...)<br>1 tajada (melón, sandía, piña...)	1 mandarina<br>3 fresas
	Hortalizas	Ensalada, verdura cruda o cocida, fresca, enlatada o congelada, guisantes, setas, gazpacho, salsa de tomate<br>NO incluye patata ni otros tubérculos	1 plato de ensalada mixta, de verduras cocinadas o en puré<br>1 tomate grande<br>2 zanahorias<br>1 vaso de gazpacho	1 zanahoria<br>2 cucharadas de salsa de tomate

Frutos secos y pipas	Frutos secos y pipas	De cualquier tipo siempre que sean tostados o naturales (nueces, almendras, cacahuetes, anacardos, pistachos, pipas de calabaza, girasol, chía, etc.) y las cremas de frutos secos/pipas sin azúcar añadido (crema de cacahuete, tahini, etc.)<br>NO incluye los frutos secos/pipas fritos/as, garrapiñadas, cubiertos de chocolate u otras coberturas, crema de cacao o de frutos secos con azúcar añadido, frutas deshidratadas	1 puñado de frutos secos/pipas ya pelados/as<br>1 cucharada sopera de crema de frutos secos sin azúcar<br>1 cucharada sopera de semillas de chía	2 nueces<br>1 cucharilla de postre de semillas
Cereales[1]	Cereales	Pan, pasta, arroz, avena<br>NO incluye cereales de desayuno comerciales, que se consideran alimentos ultraprocesados	2 trozos de pan de 4 dedos de grosor o 1 panecillo<br>1 plato hondo normal de arroz/pasta<br>4 cucharadas soperas de copos de avena	2 biscotes<br>1 plato muy grande contaría como 1 ración y 1/2
Ultraprocesados*	*Alimentos altamente procesados, ricos en azúcares, sal y/o grasa.	Galletas, bollería, repostería (tanto caseras como industriales)<br>Snacks (patatas de bolsa, chucherías, etc)<br>Postres lácteos azucarados (natillas, flan, etc)<br>Comida precocinada (pizza, lasaña, etc)<br>Etc.	6 galletas, 2 galletas de chocolate<br>1 madalena, cruasán, sobao<br>1 porción mediana de pastel<br>1 bolsa individual de patatas<br>1 porción individual de pizza, lasaña	2-3 galletas<br>1 croqueta
Grasas y aceite[2]	Grasas y aceite	Aceite de oliva virgen/virgen extra<br>Aceite de oliva, de girasol, de maíz, etc.<br>Mantequilla<br>Margarina	1 cucharada sopera	

Bebidas alcohólicas	Bebidas alcohólicas	Cerveza, vino, licores, etc.	1 botellín de cerveza<br>1 vaso de vino<br>1 chupito de licor	1 cerveza con limón contaría como 1/2 ración de cerveza y 1/2 de refresco
Refrescos y otras bebidas	Otras bebidas	Refrescos (con y sin azúcar), zumos (caseros y comerciales), bebidas energéticas, tónica, gaseosa, etc.	1 lata de refresco<br>1 vaso de zumo	
**[1]¿Cómo medir la proporción de cereales integrales de tu dieta?**				
Se trata de estimar el número de raciones que has consumido de cereales integrales (pan integral, pasta integral, arroz integral, avena, etc) respecto al total de cereales que has tomado, y reportar el resultado como un porcentaje.<br>Por ejemplo, si has tomado 1 ración de arroz blanco en la comida, 1 ración de pan blanco en la merienda, y 1 ración de pan integral en la cena, la proporción de cereales integrales será 1 (ración integral)/3 (raciones totales)=0.33 -> 33% -> 33 de 100				
**[2]¿Cómo medir la proporción de aceite de oliva virgen (extra o no) de tu dieta?**				
Se trata de estimar el número de raciones que has consumido de aceite de oliva virgen (extra o no) respecto al total de aceites/grasas (oliva, girasol, maíz, mantequilla, margarina) que has añadido a tus alimentos a lo largo del día a lo largo del día, y reportar el resultado como un porcentaje.<br>Por ejemplo, si has tomado 1 ración de mantequilla en el desayuno, 1 ración de aceite de oliva virgen en la comida, y 1 ración de aceite de oliva virgen en la cena, la proporción de aceite de oliva virgen será 2 (raciones aceite de oliva virgen)/3 (raciones totales: 1 mantequilla+2 aceite de oliva virgen)=0.66 -> 66% -> 66 de 100				
