# Supplementary material for: A Smartphone Intervention to Promote a Sustainable Healthy Diet: Protocol for a Pilot Study
Source: JMIR Res Protoc. 2023 Mar 2;12:e41443. doi: 10.2196/41443 (PMC10020902; doi:10.2196/41443)
Supplement: Multimedia Appendix 6 [file resprot_v12i1e41443_app6.pdf]

**Supplemental material 6:** Interviews guide (English and Spanish versions)

## ----English version----

### Baseline

1. *Introduction*
   - Explain the purpose of the interview, the protocol for audio recording and data storage, and the ethical principles.
   - Respond to inquiries from participants.
   - Start audio recording.

2. *Dietary habits*
   - Can you describe your typical weekday and weekend meals and where you typically eat them? (Ask questions related to the participants' regular eating habits, including when, where, with whom, and what they eat)
   - Which foods or meals are your favorite? What foods or meals do you dislike?
   - Where do you typically purchase food? For instance, grocery stores, superstores, local markets, small greengrocers or butchers, etc. What brings you to these sites most often?
   - Do you often go to restaurants? What type are they? Why do you go or not? For instance, a lack of time to prepare meals, for comfort, to try something new, etc.
   - What factors affect your diet? For example, being stressed, sad, attending social events, etc. Have you recently experienced any events that have affected your diet? Could you describe them?
   - How important are food and eating habits to your friends, roommates, and family?

3. *Healthy and sustainable diet*
   - What, in your opinion, does the term "healthy and sustainable food" mean?
   - What foods do you consider to be healthy? And which ones do you believe have low impact on the environment?
   - What should you change in your diet, in your opinion, to adopt a healthier and more environmentally friendly diet?
   - Have you tried to eat healthily?
     - If so, what strategies have you employed to try to eat healthier? What are the main obstacles you encounter?
   - Have you tried to reduce the environmental impact of your diet?
     - If so, what strategies have you employed? What were the biggest challenges you faced?
   - Farmers, collectors, store employees, and other people who work in the food production and distribution chain may not have the best or fairest working or salary conditions. Have you ever thought about this?
     - If so, do you believe you can do anything—or are you already doing something—to avoid perpetuating this circumstance?

4. *Motivations and expectations about the study*

- Are you clear about the purpose of the study and how it will be conducted? Has this purpose been clearly explained to you in the inclusion meeting?
- What appealed to you about the study and encouraged you to participate? (Ask questions related to reasons: losing weight, learning more about healthy and sustainable diets, monetary compensation, etc.)
- What do you expect to get out of your participation in the study? Do you believe the study will help you adopt a healthier and more sustainable diet? Why?

5. *Closing*
   - Is there anything we have not discussed that you would like to add?
   - Do you have questions?
   - Thank participant.

## After the intervention

1. *Introduction*
    - Explain the purpose of the interview, audio recording and data storage procedures, and ethical guidelines.
    - Answer questions from the participants.
    - Start audio recording.

2. *Perceived effectiveness of the intervention*
    - Have you observed any dietary changes since participating in the study? I refer to changes on foods consumed, food waste and also other changes related to socio-economic aspects.
        - If so, could you explain these workday and weekend changes? (Ask questions related to changes in food intake, patterns, timing, location of food consumption, where you purchase food, local products, fair trade, etc.)
        - Why do you think these changes occur? For instance, the participant is now aware of the relevance of maintaining a healthy and sustainable diet; they have recognized that it is not as difficult as they once believed it to be, due to changes in food pricing and availability, etc.
        - Is there anything concerning the study (e.g., a particular message or videocalls with the researcher explaining their progress, etc.) that has particularly called your attention or that has prompted you to make this change?
        - If not, could you please explain why you believe you have not made any changes? For instance, if the participant is not interested in changing their diet for some reason; if the study has not sufficiently motivated them to do so; if they believe it is tough to have such a diet due to their family or social environment; or if there are no dishes available at the restaurant where they usually eat.
    - Do you believe your study participation has improved your understanding of a healthy and sustainable diet? Have you learned something new? If so, what? Is there anything about this topic that you initially believed and being in the studio you have noticed that it was not so? If so, what?
    - Have you observed any changes outside your diet? For instance, how do you feel physically? Have you gained or lost weight? Have you altered your dosage if you were on medication?
    - How valuable has the study been to you overall? Have your initial expectations been met?

3. *Acceptability of the intervention*
    - Could you please describe your participation in the study? What did you like? What did you dislike? How comfortable were you with it in terms of...?
        - The number of questionnaires to be answered and the time spent on each of them; the amount or type of notifications/text messages (e.g., educational, giving advice, recipes); whether the participant finds it interesting to meet with the researcher so that they can discuss their evolution, etc.
    - Do you believe the time you dedicated to the study activities (i.e., reading the messages, completing the questionnaire, etc.) was appropriate?

- Would you recommend your family and friends to participate in the study? If so, why? If not, why not?

4. *Feasibility of the intervention*
    - Have you encountered difficulties completing your study assignments (e.g., reading text messages, filling out questionnaires, utilizing the app, etc.)? Why? or Why not?

5. *Elements to improve*
    - What, if anything, should be improved if the study is replicated with other participants?
    - How would you create an intervention to motivate participants to eat more healthfully and sustainably? What activities should the intervention include?

6. *Closing*
    - Is there anything we have not discussed that you would like to add?
    - Do you have questions?
    - Thank participant.

**Interview guide (Follow-up)**

1. *Introduction*
   - Explain the purpose of the interview, audio recording and data storage procedures, and ethical guidelines.
   - Answer questions from the participants.
   - Start audio recording.

2. *Maintenance: facilitators, barriers*
   - We have noted that after we discontinued sending you notifications, and ceased holding meetings, you have maintained/changed your diet over time. What do you think the reason is? (i.e., examine barriers and facilitators to the maintenance of the eating behaviors acquired during the intervention, based on the results of each participant)
   - (If the changes identified during the intervention have been maintained over time or the diet has improved). Do you believe your improved diet is due to your participation in the study? If yes, for what specific purpose? For instance, a particular message caught your attention and made you aware of the importance of a healthy and sustainable diet, etc.

3. *Closing*
   - Is there anything we have not discussed that you would like to add?
   - Do you have any questions?
   - Thank participant.

**----Spanish version----**

**Comienzo:**

1. *Introducción*
   - Explicación del propósito de la entrevista, de los procedimientos de grabación de audio y almacenamiento de datos y de las pautas éticas.
   - Respuesta a las preguntas de los participantes.
   - Inicio de la grabación.

2. *Comportamiento alimentario*
   - ¿Puede describir qué suele comer y dónde lo hace normalmente, tanto entre semana como durante los fines de semana? (Preguntas adicionales sobre cuándo, dónde, con quién y qué come típicamente el participante)
   - ¿Cuáles son sus comidas o alimentos favoritos? ¿Y qué comidas o alimentos le disgustan?
   - ¿Dónde suele comprar la comida? Por ejemplo, en supermercados, hipermercados, mercado del barrio, pequeñas fruterías o carnicerías, etc. ¿Por qué suele ir a estos sitios?
   - ¿Va frecuentemente a restaurantes? ¿De qué tipo son? ¿Por qué va o no va? Por ejemplo, por falta de tiempo para cocinar, por comodidad, por probar cosas nuevas, etc.
   - En su opinión, ¿qué factores influyen en su alimentación? Por ejemplo, estar estresado, triste, eventos sociales, etc. ¿Ha experimentado recientemente algún hecho en particular que haya afectado a su alimentación? ¿Podría describirlo?
   - ¿Qué importancia le dan a la alimentación y a la elección de alimentos los miembros de su familia, sus compañeros de piso o sus amigos?

3. *Alimentación saludable y sostenible*

- ¿Qué significa para usted el concepto "alimentación saludable y sostenible"?
- ¿Qué alimentos cree que son saludables? ¿Y cuáles cree que tienen un impacto ambiental reducido?
- ¿Qué cree que debe cambiar de su alimentación para seguir una dieta más saludable y con menor impacto ambiental?
- ¿Intenta comer saludable?
  - Si es así, ¿Qué estrategias utiliza para intentar comer más saludablemente? ¿Cuáles son los principales obstáculos que encuentra?
- ¿Ha intentado reducir el impacto ambiental de su alimentación?
  - Si es así, ¿Qué estrategias ha seguido? ¿Cuáles son los principales obstáculos que encuentra?
- Muchas veces, las condiciones laborales y salariales de las personas implicadas en la cadena de producción y distribución de alimentos (por ejemplo, los agricultores, recolectores, personal de las tiendas, etc.) no son las mejores, ni tan siquiera justas. ¿Se ha parado a pensar alguna vez sobre esto?
  - Si es así, ¿cree que puede hacer algo, o hace algo, para evitar perpetuar esta situación?

4. *Motivaciones y expectativas del estudio*

- ¿Tiene claro el propósito del estudio y cómo se llevará a cabo? ¿Se lo han explicado claramente en la reunión?
- ¿Qué le atrajo del estudio y le animó a participar? (Preguntar por motivos: perder peso, aprender más sobre dietas saludables y sostenibles, la remuneración económica, etc.)
- ¿Qué espera obtener de su participación en el estudio? ¿Cree que le ayudará a cambiar su alimentación hacia una más saludable o sostenible? ¿Por qué?

5. *Cierre*
   - ¿Hay algo que no hayamos discutido que le gustaría agregar?
   - ¿Tiene preguntas?
   - Dar las gracias al participante.

**Final de la intervención:**

1. *Introducción*
    - Explicar el propósito de la entrevista, de los procedimientos de grabación de audio y almacenamiento de datos y de las pautas éticas.
    - Respuesta a las preguntas de los participantes.
    - Inicio de la grabación.

2. *Eficacia percibida de la intervención*
    - ¿Ha notado algún cambio en su alimentación desde que se encuentra participando en el estudio? Tanto en los alimentos que consume, como en el desperdicio de comida, así como cambios relacionados con aspectos más socio-económicos.
        - *En caso afirmativo*, ¿podría explicar esos cambios tanto para los días laborables como para el fin de semana? (Preguntas adicionales sobre los cambios en los alimentos consumidos, los patrones, el momento y la ubicación del consumo de alimentos, dónde los compra, productos locales, comercio justo, etc.)
        - ¿A qué cree que se deben esos cambios? Por ejemplo, ha tomado conciencia de la importancia de seguir una alimentación saludable y sostenible, se ha dado cuenta de que no era tan complicado, por cambio de precios o de la disponibilidad de algunos alimentos, etc.
        - ¿Hay algo del estudio, algún mensaje en concreto, o las llamadas del investigador para explicarle su evolución, etc. que le haya llamado especialmente la atención, o que le haya resultado más interesante, o le haya motivado a dar ese cambio?
        - *En caso negativo,* ¿por qué cree que no ha hecho ningún cambio? Por ejemplo, no le interesa cambiar su alimentación por algún motivo, el estudio no le ha motivado lo suficiente como para cambiar, considera que es muy difícil llevar una alimentación así por el entorno familiar o social, no hay disponibilidad de platos en el restaurante donde suele ir a comer…
    - ¿Cree que estar en el estudio le ha permitido conocer mejor en qué consiste una alimentación saludable y sostenible? ¿Ha aprendido? Si es así, ¿el qué? ¿Hay algo sobre este tema que inicialmente creía y estando en el estudio se ha dado cuenta de que no era así? Si es así, ¿el qué?
    - Más allá de la alimentación, ¿ha notado algún cambio? Por ejemplo, se siente mejor o peor físicamente, ha subido o bajado peso, ha cambiado su medicación si es que tomaba, etc.
    - En cómputo general, ¿Qué tan útil fue el estudio para usted? ¿Se han cumplido sus expectativas iniciales?

3. *Aceptabilidad de la intervención*
    - ¿Puede describir su experiencia en el estudio? ¿Qué le gusta? ¿Qué le disgusta? ¿Qué tan cómodo se siente en él? Por ejemplo:
        - el número de cuestionarios a responder, o tiempo que dedica a cada uno de ellos,
        - el número o el tipo de mensajes (informativos, dando consejos, recetas),

    - si considera interesante o no tener la reunión con el investigador para que comenten su evolución, etc.
- El tiempo que le ha dedicado al estudio (leer los mensajes, responder el cuestionario, etc.), ¿le parece adecuado?
- ¿Recomendaría a su familia y amigos que participasen en él? Si es así ¿por qué? Si no, ¿por qué no?

4. *Factibilidad de la intervención*
    - ¿Ha experimentado alguna dificultad para completar sus tareas en el estudio (es decir, leer los mensajes de texto, completar los cuestionarios, usar la aplicación, etc.)? Si es así, ¿por qué? Si no, ¿por qué no?

5. *Aspectos a mejorar*
    - ¿Qué debería mejorarse, si es que hay algo, en el estudio si queremos volver a realizarlo con otros participantes?
    - ¿Cómo cree que se podría diseñar una intervención para motivar a los participantes a comer de manera más saludable y sostenible? ¿Qué actividades deberían incluirse en la intervención?

6. *Cierre*
    - ¿Hay algo que no hayamos discutido que le gustaría agregar?
    - ¿Tiene preguntas?
    - Dar las gracias al participante.

**Fin del estudio:**

1. *Introducción*
   - Explicar el propósito de la entrevista, de los procedimientos de grabación de audio y almacenamiento de datos y de las pautas éticas.
   - Respuesta a las preguntas de los participantes.
   - Inicio de la grabación.

2. *Mantenimiento: facilitadores, barreras*
   - Hemos detectado que desde que dejamos de mandarle mensajes y de hacer las reuniones de seguimiento con usted, usted… (exponer si ha mantenido su alimentación durante el tiempo o la ha cambiado), ¿a qué cree que se debe? (explorar facilitadores y barreras en función de los resultados de cada participante)
   - (Si los cambios obtenidos durante la intervención se han mantenido en el tiempo o incluso la alimentación ha mejorado). ¿Cree que esta mejora en su alimentación se debe a su participación en el estudio? Si es que sí, ¿por alguna cosa en concreto? Algún mensaje que le llamó la atención en concreto, tomó conciencia de la importancia de seguir una alimentación saludable y sostenible, etc.

3. *Cierre*
   - ¿Hay algo que no hayamos discutido que le gustaría agregar?
   - ¿Tiene preguntas?
   - Dar las gracias al participante.
